# Hexagonal Hybrid Bismuthene by Molecular Interface Engineering

**DOI:** 10.1021/jacs.2c13036

**Published:** 2023-06-01

**Authors:** Christian Dolle, Víctor Oestreicher, Alberto M. Ruiz, Malte Kohring, Francisco Garnes-Portolés, Mingjian Wu, Gabriel Sánchez-Santolino, Alvaro Seijas-Da Silva, Marta Alcaraz, Yolita M. Eggeler, Erdmann Spiecker, Josep Canet-Ferrer, Antonio Leyva-Pérez, Heiko B. Weber, María Varela, José J. Baldoví, Gonzalo Abellán

**Affiliations:** † Instituto de Ciencia Molecular (ICMol), Universidad de Valencia, Catedrático José Beltrán Martínez n° 2, 46980 Paterna, Spain; ‡ Lehrstuhl für Angewandte Physik, Friedrich-Alexander Universität Erlangen-Nürnberg (FAU), Staudtstr. 7, 91058 Erlangen, Germany; § Instituto de Tecnología Química (UPV−CSIC), Universitat Politècnica de València−Consejo Superior de Investigaciones Científicas, Avda. de los Naranjos s/n, 46022 Valencia, Spain; ∥ Institute of Micro- and Nanostructure Research (IMN) & Center for Nanoanalysis and Electron Microscopy (CENEM), Interdisciplinary Center for Nanostructured Films (IZNF), Friedrich-Alexander Universität Erlangen-Nürnberg (FAU), Cauerstraße 3, 91058 Erlangen, Germany; ⊥ Instituto Pluridisciplinar & Departamento de Física de Materiales, Universidad Complutense de Madrid (UCM), Plaza de Ciencias 1, Ciudad Universitaria, 28040 Madrid, Spain; # Laboratory for Electron Microscopy (LEM), Microscopy of Nanoscale Structures & Mechanisms (MNM), Karlsruhe Institute of Technology (KIT), Wolfgang-Gaede Str. 1a, 76131 Karlsruhe, Germany

## Abstract

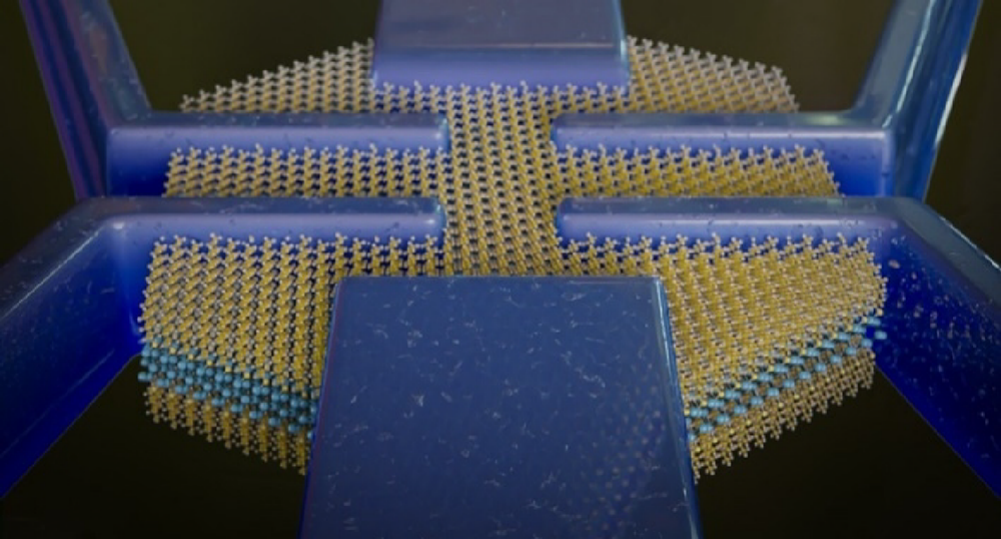

High-quality devices based on layered heterostructures are typically built from materials obtained by complex solid-state physical approaches or laborious mechanical exfoliation and transfer. Meanwhile, wet-chemically synthesized materials commonly suffer from surface residuals and intrinsic defects. Here, we synthesize using an unprecedented colloidal photocatalyzed, one-pot redox reaction a few-layers bismuth hybrid of “electronic grade” structural quality. Intriguingly, the material presents a sulfur-alkyl-functionalized reconstructed surface that prevents it from oxidation and leads to a tuned electronic structure that results from the altered arrangement of the surface. The metallic behavior of the hybrid is supported by *ab initio* predictions and room temperature transport measurements of individual nanoflakes. Our findings indicate how surface reconstructions in two-dimensional (2D) systems can promote unexpected properties that can pave the way to new functionalities and devices. Moreover, this scalable synthetic process opens new avenues for applications in plasmonics or electronic (and spintronic) device fabrication. Beyond electronics, this 2D hybrid material may be of interest in organic catalysis, biomedicine, or energy storage and conversion.

## Introduction

The field of two-dimensional (2D) materials has blossomed in the past decades due to the extraordinary properties that these materials show upon reduction of their thickness.^1^ After sophisticated studies of graphene,^2^ the focus of research started to pivot toward heavier layered materials with inherent promising electronic properties, such as MXenes, and transition metal dichalcogenides (TMDCs).^3,4^ At the frontier of this trend, the group of 2D pnictogens (group 15 of the periodic table) stands out as one of the most appealing, with phosphorene being the first obtained pnictogen that allowed to build field-effect transistors (FETs) with high on/off ratios and excellent mobilities.^5−62^ Furthermore, one exciting property for the heavy pnictogens, and especially antimony and bismuth, is a strong spin–orbit coupling (SOC) that is crucial for achieving topological surface states and spin orbit torque (SOT) effects, thus opening the door for exotic quantum phenomena and unprecedented opportunities for 2D spintronics and magnonics.^8−10^ Also, the catalytic activity^11^ of this cost efficient material as well as the extremely low toxicity may lead to a wide variety of applications in the foreseeable future.^12−15^

However, one of the drawbacks in the preparation of this material is the considerable covalent character of the interlayer bonding, an effect that increases with the atomic weight and thus the resulting SOC of the pnictogen, precluding their exfoliation. Moreover, this strong interlayer interactions generally lead to a decrease in lateral sizes, poor morphologies, and pronounced oxidation^15,16^ upon mechanical top-down production methodologies. In turn, complex and technologically high-demanding gas-phase bottom-up approaches do not allow controlling the morphology and are still in the development phase.^17^ When it comes to the heaviest pnictogen bismuthene, the production of 2D materials exhibiting controlled morphologies, large lateral dimensions, low degrees of oxidation, and high optical and electrical qualities remains an open challenge.^18^ Moreover, wet chemical approaches usually generate contaminated surfaces,^19^ hampering the general processing and the construction of microdevices from the synthesized materials, thus impeding their appropriate electrical characterization.

In this work, we challenge the common belief that wet chemical synthesis of high-quality 2D materials is technologically unfeasible, showing that it is possible to produce a large and thin bismuth system that can act as a technological platform, employable in studying quantum phenomena. The material is formed by ultrathin (∼5 nm) and large (diameter > 1 μm) hexagonal bismuth sandwiched single crystals, showing appealing optical and electronic properties. This material exhibits a particular structure: a solid core of few-layers β-bismuth, with a surface reconstruction consisting of sulfur-functionalized layers on both basal planes. The hexagonal bismuthene interfaces have an enlarged, yet commensurate, in-plane lattice parameter and behave as metallic conductors at room temperature, giving rise to a hybrid heterostructure with a semi-metallic core and high SOC. This results in a potential candidate to produce current-induced SOT, thus influencing the magnetization and spin dynamics of an interfaced magnetic material. This approach allows the synthesis of 2D hexagonal hybrid bismuth nanomaterials with protected surfaces under environmental conditions, thus offering an excellent platform for the study of experimental physics, as well as a scalable pathway for exploring applications in organic catalysis, biomedicine, plasmonics, or energy storage and conversion, to name a few.

## Results and Discussion

### Synthesis and Characterization

**Figure 1:**
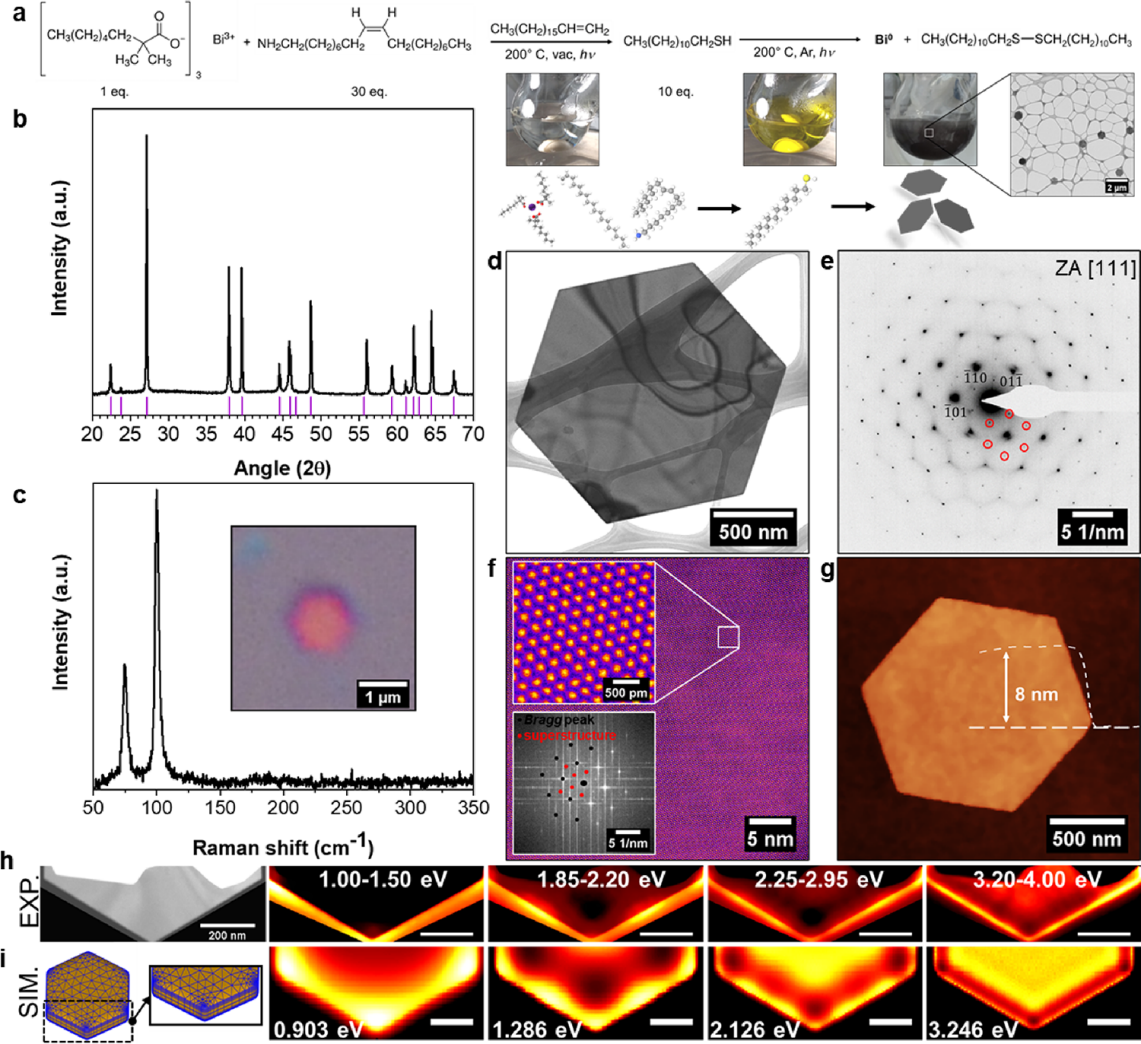
Colloidal synthesis and structural investigation of the bismuth/bismuthene heterostructure. (a) Reaction equation for the colloidal synthesis of the bismuth/bismuthene sandwich material with given molar equivalents for the reaction. Photographs show the progressing color change along the reaction coordinate during the reduction of Bi^III^ to Bi^0^. (b) Powder X-ray diffraction (PXRD) pattern of the synthesized material with the expected diffraction peaks indicated by pink ticks. (c) Typical unprocessed Raman spectrum of individual crystallite. No indication of surface oxidation is noticeable. The inset shows the optical micrograph of individual crystallite. (d) Overview bright field transmission electron microscopy (BF-TEM) data and (e) typical indexed diffraction pattern of the hybrid material with superstructure reflections indicated. (f) Colorized large area high-resolution scanning transmission electron microscopy (HRSTEM) data with the blown-up area and FFT (with contributions from Bragg and superstructural reflections indicated as black and red dots, respectively) as the inset. No local defects are visible in the data. (g) AFM of individual crystallite with indicated height profile. (h) Experimental series of integrated electron energy-loss spectroscopy (EELS) intensity in the given energetic ranges, showing localized surface plasmon modes and (i) comparative simulation of plasmonic modes based on presented model hexagon. Comparative EEL spectra contained in Supplementary Figure 3.

The synthetic route to obtain the hexagonal, single-crystalline, 2D surface reconstructed bismuth is based on a colloidal, photocatalyzed, one-pot redox reaction, as shown in Figure 1a (see also Supplementary Figure 1). Herein, the Bi^III^ precursor bismuthneodecanoate (BiNeo) is reduced by dodecanethiol (DDT) under visible light illumination, yielding 2D bismuth/bismuthene hybrids.^20,21^ In the redox reaction, the employed thiol is oxidized to form a disulfide^22^ and the process can be followed by a color change of the reaction mixture from colorless (Bi^III^) to yellow (after the addition of the reductant DDT and the formation of Bi(SR)_3_) to a finally black suspension (see photographs in Figure 1a and Supplementary Movie SM1; details given in the Methods Section). The generated crystallites are isolated from the reaction mixture by centrifugation and washed with chloroform before drying the obtained solid in an inert gas glovebox. The final solid black product shows the expected rhombohedral structure in powder X-ray diffraction (PXRD), as evident from Figure 1b, while Raman spectroscopy (Figure 1c), due to the absence of a signal between 300 and 350 cm^–1^, indicates no prominent oxidation and exclusively presents the fundamental vibrations with E_g_ and A_1g_ symmetry at 75 and 102 cm^–1^, respectively.^23,24^ A pronounced thickness dependence for the fundamental vibrations is observed (see Supplementary Figure 2), allowing for a thickness classification based on Raman spectroscopy.^25,26^ The inset of Figure 1c furthermore shows an optical micrograph of one individual bismuth/bismuthene hybrid crystallite on a silicon substrate with a typical pinkish color, indicating a thickness below 10 nm. Further microscopic investigation reveals defined hexagonal particles (Figure 1d,g), with a low thickness to aspect ratio (here, 8 nm thickness *vs* 1.5 μm diameter), classifying the material as a true 2D structure. High-angle annular dark-field high-resolution scanning transmission electron microscopy (HAADF-HRSTEM) in Figure 1f does not show any local defects on the atomic scale but, interestingly, exhibit a superstructure, associated with an additional crystalline ordering, as indicated by the extra spots in the fast Fourier transform (FFT) of the respective micrograph shown as the inset. This additional superstructure is furthermore contained in the selected area electron diffraction (SAED) pattern of individual crystals, as shown in Figure 1e, and originates from the hybrid superstructure, as explained below.

### Localized Surface Plasmon Resonances

The structural characterization is complemented by low-loss EEL spectrum imaging (see Figure 1h). The presented data were acquired by aberration-corrected and monochromated STEM-EELS and compared to numerical simulations in Figure 1i (and Supplementary Figure 3), showing four distinct localized surface plasmon resonances (LSPRs). This offers an additional proof of the materials’ quality based on the agreement between experiments and simulations for the observation of LSPRs in the short-wavelength IR range. On the one hand, the linewidth of LSPRs is intrinsically connected to the materials’ quality^27−29^ and the observation of LSPRs in ultrathin metals (≤10 nm thick) is extremely challenging. This is first, due to the increase in the electron damping by scattering at the interfaces and surface defects and, second, because ultrathin nanostructures are extremely sensitive to surface roughness. On the other hand, thin bismuth films are very difficult to grow by means of physical vapor deposition methods and the determination of the dielectric function of bismuth is strongly affected by the sample quality.^30^ The best fitting to our experimental data in the EELS simulations is found by using the dielectric function of bismuth reported by Lenham *et al*.,^31^ which was measured on a polished single crystal of bismuth. Identical EELS maps (and very similar EEL spectra) are simulated using the dielectric function published by Toudert *et al.*,^30^ which provides a Kramers–Kroning consistent dielectric function by modeling the surface roughness. Numerical solutions based on other dielectric functions available in the literature show that the LSPRs are dramatically broadened or quenched.

It is also worth mentioning that to our knowledge, LSPRs based on ultrathin metals have been only achieved in silver and gold, grown by means of specialty methods, and used for very specific applications.^27,32^ Hence, the observation of LSPRs in a resistive semimetal-like bismuth is especially surprising and only possible for high-quality samples. On top of that, our synthetic route is based on a purely wet-chemical approach without any post-preparation treatments and allows for exploring the plasmonic and photocatalytic activity arising from a strong interband absorption in bismuth.^30^ The individual bismuth flakes even can be isolated and directly submitted to nanofabrication processes (like transport measurements, see below), allowing the fabrication of ultrathin optoelectronic devices from a purely chemical synthetic approach.

### Surface Functionalization and Hybrid Superstructure

**Figure 2:**
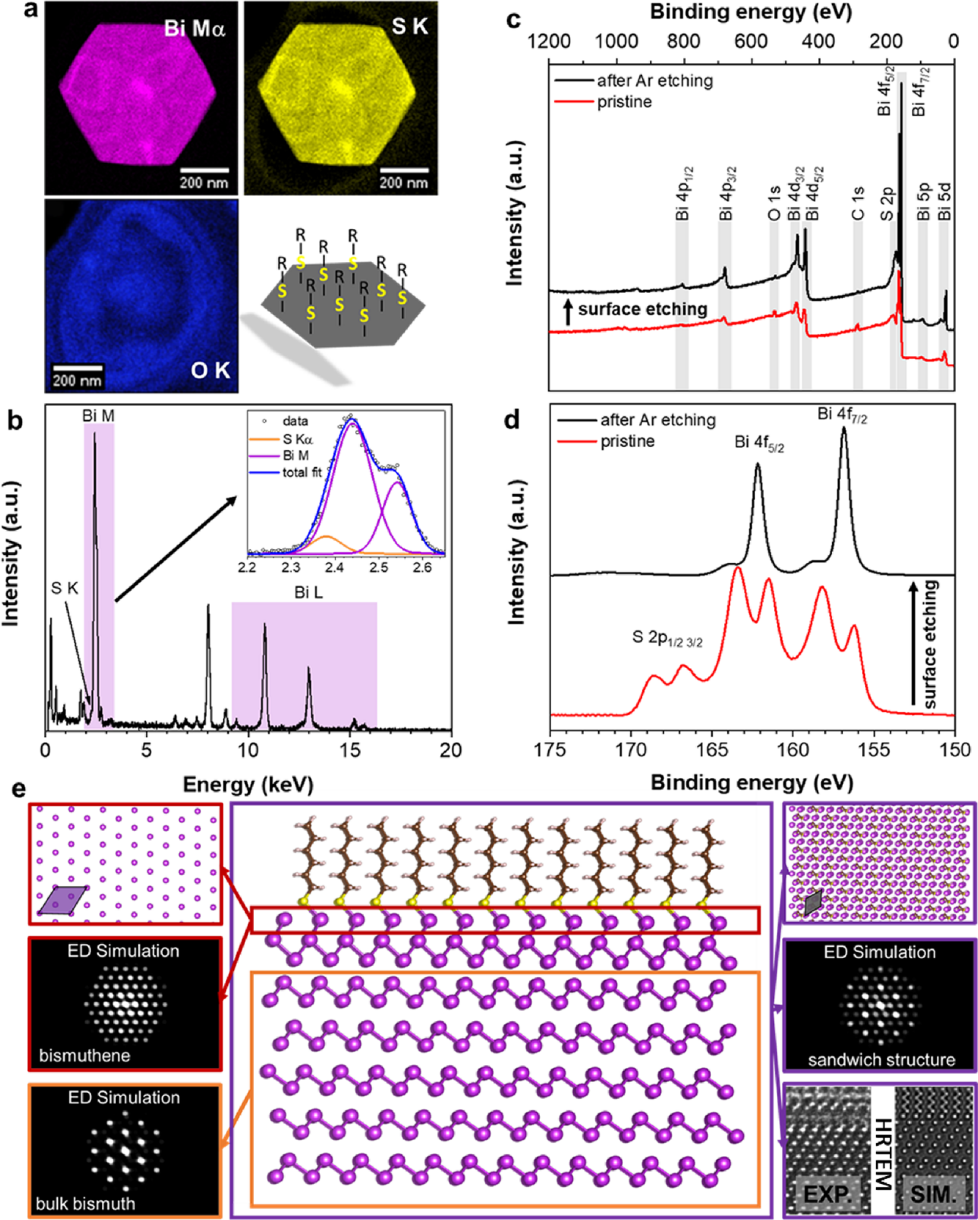
Surface composition and hybrid structural model. (a) Integrated scanning transmission electron microscopy energy-dispersive X-ray spectroscopy (STEM-EDX) intensity for the Bi-Mα, S-K, and O-K signals for individual crystallite with artistic representation of the functionalized material. (b) Spatially integrated mean EDX spectrum with signals related to Bi indicated. Additional peaks can be ascribed to stray radiation from the sample support and holder. The inset shows a close-up of the energy range between 2.2 and 2.7 keV, with a fitting of the minute sulfur contribution resulting from the functionalized surface. (c) Overview X-ray photoemission spectroscopy (XPS) spectrum before (red) and after (black) surface etching with Ar ions and (d) high-resolution XPS of S/Bi binding energy range. A clear indication of S 2p before the surface etching can be noticed between 165 and 170 eV (detailed fit of individual contributions in Supplementary Figures 5 and 6). (e) Structurally optimized bismuth/bismuthene hybrid with simulated electron diffraction patterns from the indicated regions. The bottom right panel shows a cross-sectional HRTEM image and the respective image simulation by a multislice approach. Cross section prepared by FIB milling (Supplementary Figure 9). Additional cross-sections and detailed analyses can be found in Supplementary Figure 10.

Next, a closer look at the interfaces will help to unravel the chemical and structural peculiarities and to address the observed superstructure and the impact of the interfaced hybrid material on its expected electronic behavior. First, a STEM-EDX mapping is shown in Figure 2a. The integrated intensities of Bi-Mα, S-K, and O-K peaks are shown together with a sketch of the postulated thiol surface functionalization. Based on the oxygen map, no prominent oxidation is expected (only the TEM substrate contains oxygen), while a closer look at the Bi-M signal reveals a minute contribution of sulfur on the low energy side of Bi-Mα, as presented in the inset of Figure 2b. Due to a strong overlap between Bi-M and S-K, we did not attempt a direct quantification of the amount of sulfur but, by following the time-dependent relative atomic percentage of sulfur and bismuth content, an indication for an electron beam induced defunctionalization of the material can be observed (see Supplementary Figure 4). To avoid electron beam-induced damage and focus on a more surface-sensitive technique to qualitatively study the observed sulfur contribution, we chose X-ray photoelectron spectroscopy (XPS). In Figure 2c,d, the respective survey and the high-resolution XPS spectra of the S/Bi core energies of a typical batch of the bismuth hybrid are shown. Here, the red curve is the pristine material with a strong overlap of S and Bi, but with the S p orbital, energies clearly separated as individual signals between 170 and 165 eV. Detailed fits of the binding energy ranges of Bi, O, and C are contained in Supplementary Figures 5–7. The relatively high binding energy of sulfur points toward a strong influence of the underlying bismuth on the involved sulfur species,^33,34^ which hints to a covalent attachment of sulfur to the terminating bismuth layers (see also Supplementary Figure 5). This covalent anchoring also influences the oxidation state of Bi on the materials’ surfaces, reflected in the shift of binding energy toward Bi^III^, as seen in the pristine signal of the hybrid material (red trace) in Figure 2d. It is worth mentioning that the observed sulfur contribution is only localized directly at the outer surfaces of the crystallites and not contained within the material. This is corroborated after etching the material in the vacuum of the XPS with a focused Ar ion beam for 30 s, thus sputtering the outer surfaces (details in the Methods section). After the applied sputtering, only an almost pure bismuth signal can be recorded in the high resolution XPS spectrum, while the contributions of sulfur, carbon, and oxygen are practically negligible (detailed fits in Supplementary Figures 6 and 7). A small signal related to Bi^III^ after surface etching in Figure 2d (black trace) can be attributed to the statistical distribution of crystallites and the relative angle of the sputtering source that does not access all free surfaces in the etching process, thus leaving either small areas with a remaining sulfur functionalization or adsorbed sulfur on the surfaces that contribute to the presented XPS signal. To exclude the oxidation of the hybrid material, we compared the electron diffraction pattern of freshly synthesized samples to a sample batch that was stored under ambient conditions for several months. Besides the superstructural reflections, in addition to the expected Bragg peaks (see Figure 1e), the oxidation of the outer layers of the hybrid material to Bi_2_O_3_ is reflected in the appearance of new signals, presented in Supplementary Figure 8. To rationalize the possible formation of a sulfur-functionalized surface layer in the synthesis process, we conceived the hybrid structure model, shown in Figure 2e, and performed density functional theory (DFT) calculations to check for its theoretical stability. The model consists of an inner “bulk” part of β-Bi with the typical zig-zag arrangement of bismuth, terminated by flat hexagonal layers of bismuthene with a lattice constant of 7.5379 Å after structural optimization. Here, the surface layer in the model is covalently fully functionalized with hexylthiol groups to stabilize the larger lattice constant. Specifically, the hybrid was set up by stacking six layers of rhombohedral Bi and terminating it with a half-layer of in-plane arranged Bi atoms that are covalently bonded to hexylthiol; the structure was then fully relaxed by means of DFT with dispersion interactions and checked for stability (for details, see the Methods Section and the Supporting Information). We used this hybrid structure for the following theoretical calculations of physical properties and simulation of microscopic data, and benchmark this hypothesis against our experimental findings.

With the structurally stable atomic model at hand, we were able to simulate electron diffraction patterns in different arrangements to compare them to the experimental results. The simulated electron diffraction patterns of the pure β-bismuth and the half-layer bismuth section along [111] are shown in Figure 2e in orange and red, respectively. The 30° rotation between the two domains, as observed in the experimental diffraction pattern (see Figure 1e), and also in the simulation of the hybrid stack, depicted in purple, is intriguing. Furthermore, in the atomic arrangement of the terminating bismuthene layer, when observed along the [111] direction, a much larger lattice constant (indicated by the purple diamond), can be noticed. It is worth mentioning that this novel, flat structure is a direct consequence of the covalent stabilization by the employed thiol and, in practice, only achievable by our chemical approach. To clarify the obtained structure, we extracted cross-sectional FIB lamellas. On the lower right of Figure 2e, the direct comparison of experimental HRTEM data and a simulated image based on the model is shown (details in the Supporting Information and Supplementary Figure 9), locally obtaining a good match between experimental HRTEM data and image simulation. Moreover, the high-resolution high-angle annular dark-field (HAADF) scanning transmission electron microscopy cross-sectional images of the edge of different bismuth flakes reveal a 1–2 nm-thick reconstructed layer on the top and bottom surfaces of the flake and also on the edge on the side (Supplementary Figure 10). The STEM-EDX mappings show the postulated subtle sulfur contribution on the materials’ surfaces (see Supplementary Figure 11). Additionally, the STEM-EELS maps of the cross section show a slight oxidation of the surface reconstructed layers, along with some carbon presence related to surface contamination (Supplementary Figure 12). Finally, a focal series of HAADF-HRSTEM images (equivalent to performing a depth-sectioning experiment) acquired along the β-Bi [111] direction (Supplementary Figure 13 and Supplementary Movie SM2) supports the structural transition from the superficial hybrid bismuth/bismuthene structure to the internal β-Bi, excluding the encapsulation between bismuth oxide layers. In atomic resolved ac-HRSTEM investigations of cross-sectional lamellas, we furthermore found arrangements of isolated buckled bismuthene monolayers separated from the “bulk” by 4.56 Å (see Supplementary Figure 14). While the observed structural arrangement can also be in agreement with electron diffraction, we address the local establishment of lifted bismuthene patches to the result of an electron beam-induced defunctionalization of the terminating layers during preparation of the FIB lamella (see also Supplementary Figure 4).

### Catalytic Results

**Figure 3:**
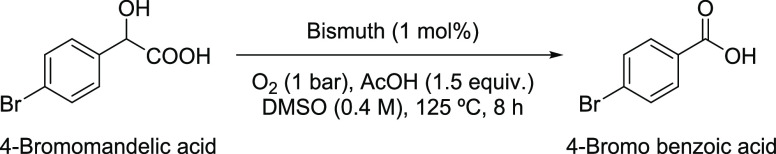
Catalytic oxidation reaction under bismuth catalysis.

Catalysis is a surface phenomenon;^35^ thus, a catalytic probe reaction will allow us to further confirm the protection of the surface, exerted by the thiol groups on the 2D hybrid material. Table 1 shows the catalytic results for the decarboxylative oxidation reaction of 4-bromomandelic acid to 4-bromo benzoic acid (see Figure 3), which has been reported to be catalyzed by bulk Bi^0^ metal under aerobic conditions at 125 °C, in acetic acid/dimethyl sulfoxide (DMSO) as a solvent mixture.^36^ These reaction conditions are sufficiently harsh to remove any thiol ligand not strongly coordinated to the bismuthene surface and oxidize the material to bismuth oxide (Bi_2_O_3_), and thus, it can be used as a probe reaction to assess if thiols are really covering and protecting the surface of the 2D bismuth hybrid material.^37^ The results show that both Bi^0^ metal and Bi_2_O_3_ are competent catalysts for the reaction at 1 mol % to give ∼80% yield of 4-bromo benzoic acid after 8 h reaction time (entries 1 and 2). Conversion of the starting material was complete, and the mass balance is closed with some partially oxidized products. The similar product yields obtained for the Bi^0^ metal and Bi_2_O_3_ catalysts suggest that Bi^0^ is oxidized to Bi_2_O_3_ under the strong oxidizing reaction conditions.

**Table 1:** Catalytic Results^a^

entry	catalyst	time in air (months)	yield (%)
1	Bi^0^		84
2	Bi_2_O_3_		78
3	unprotected bismuth particles	0	72
4		3	70
5	2D sandwiched bismuth/bismuthene hybrid	0	4
6		3	5

a Decarboxylative oxidation reaction of 4-bromo mandelic acid to 4-bromo benzoic acid catalyzed by 1 mol % of different bismuth species under the above indicated reaction conditions. Gas chromatography yields. Total conversion of the starting material is found in entries 1–4, and mass balances are completed with partially oxidized products. Selectivity for the product is complete in entries 5 and 6 (no more products found).

Table 1 also shows that pristine spherical bismuth particles (see Supplementary Figure 1A), added into the reaction flask in the glovebox, act as an active catalyst for the probe reaction, to give 72% of the product, after total conversion (entry 3). To our knowledge, this is the first thermal organic reaction catalyzed by pristine bismuth.^38^ When the unprotected material was left to the open air for 3 months, outside of the glovebox, and was used as a catalyst for the reaction, the result was practically the same (70%, entry 4). These results are in good agreement with the similar catalytic activity found for Bi^0^ metal (84%) and Bi_2_O_3_ (78%). In contrast, the hybrid material did not show any catalytic activity neither when added fresh into the reaction, in the glovebox, or after 3 months at the open air. In both reactions, the yield of product 4-bromo benzoic acid is very low (<6%), without any other product found (conversion also <6%). In other words, the 2D hybrid material possesses protecting groups on the surface (thiols) that do not allow the reagents to reach the bismuth atoms, and thus the catalytic reaction does not occur. It is worth to comment here that the aerobic conditions of the probe catalytic reaction do not trigger thiol oxidation, which would require stronger oxidants such as bleach or peroxides.

To confirm the suppression of catalytic activity by the surface thiol groups of the hybrid material, a second probe reaction was found and tested. This second reaction consists of a Friedel–Crafts acylation reaction between benzoyl chloride and toluene, performed at 150 °C with the co-catalysis of a very polar organic molecule, *i.e.*, the ionic liquid ethylmethylimidazolium triflimide [emim][NTf_2_].^39^ These reaction conditions, although different from those in the first catalytic probe reaction, are still harsh enough to remove any thiol, which is not strongly joint to the 2D material. The results (Supplementary Table 1) show that unprotected bismuth particles approach the catalytic activity of Bi_2_O_3_ (∼80% yield of product) after the pristine material is left to the open air and thus oxidized. In contrast, the thiol-protected hybrid material requires 6 months outside the glovebox, at the open air, to reach just a 12% of Friedel–Crafts products. These results further support the extremely low oxidation (absence of BiO_*x*_, for instance Bi_2_O_3_) experienced by the 2D hybrid material at the open air and the lack of catalytic activity.

We then performed the XRD, XPS, and Raman measurements of the spent hybrid material after the decarboxylative oxidation reaction of 4-bromo mandelic acid to 4-bromo benzoic acid (Figure 3 above), where our protected material is not active, while Bi_2_O_3_ and Bi(0) indeed are (the latter by in situ oxidation). First, we performed a capillary XRD experiment, where the new bismuthene material was recorded on the same reaction mixture, without extracting the solid material, to avoid any further oxidation or interference by a separation method. The result (Supplementary Figure 15) shows that the dispersed and surface XRD of the used hybrid material are mainly the same (compare blue lines above and black lines below), and thus both measurements are equivalent, which allows us to now compare it with the surface XRD of bulk Bi_2_O_3_ and Bi(0). It can be seen, despite the weakness of the signals due to the dispersion of the material in the reaction mixture, that the XRD of the spent hybrid material still shows the main peaks of the original material at 2θ = 27 and 32° (see Figure 1b, above), which are completely different to Bi_2_O_3_ and Bi(0). These results suggest that the hybrid material mainly keeps its structure after reaction.

Once we know that the spent hybrid material can be isolated from the reaction mixture without severe modification of its structure, we performed Raman and XPS measurements. The Raman spectrum (Supplementary Figure 16) shows the two original peaks at ∼75 and 105 cm^–1^ (see Figure 1c, above), together with some peaks that may fit well with Bi(0) particles. However, the formation of Bi_2_O_3_ cannot be detected, *i.e*., by the lack of signal at ∼130 cm^–1^. The corresponding XPS spectra (Supplementary Figure 17), either at 200 or 400 μm spot sizes, show the S 2p peaks together with the Bi 4f signals (see survey) and the complex deconvoluted signals for C 1s and O 1s, as in the original material (*i.e.*, see Figure 2c, above). These results are in accordance with the XRD results and together indicate a low degree of degradation of the hybrid material during reaction, in good agreement with its lack of catalytic activity for the reaction and in contrast to bulk Bi_2_O_3_ and Bi(0).

The unique catalytic activity of a particular crystal plane of Bi(0), not present in the hybrid material, may be possible, however, quite unlikely. Metallic Bi particles made from the same experimental route as well as commercially bought bulk Bi metal have all statistically relevant lattice planes accessible during reaction, including the basal plane mainly present in the hybrid material, and only the Bi(0) materials without thiol protection are catalytically active. In fact, the catalytic reaction balance is highly comparable for the two ligand-free Bi(0) materials. In other words, it is difficult at this point to accept any direct correlation between accessible lattice planes/preferential orientations and the catalytic activity of the material (at least under the present experimental conditions).

### Electronic Properties and Transport Measurements

**Figure 4:**
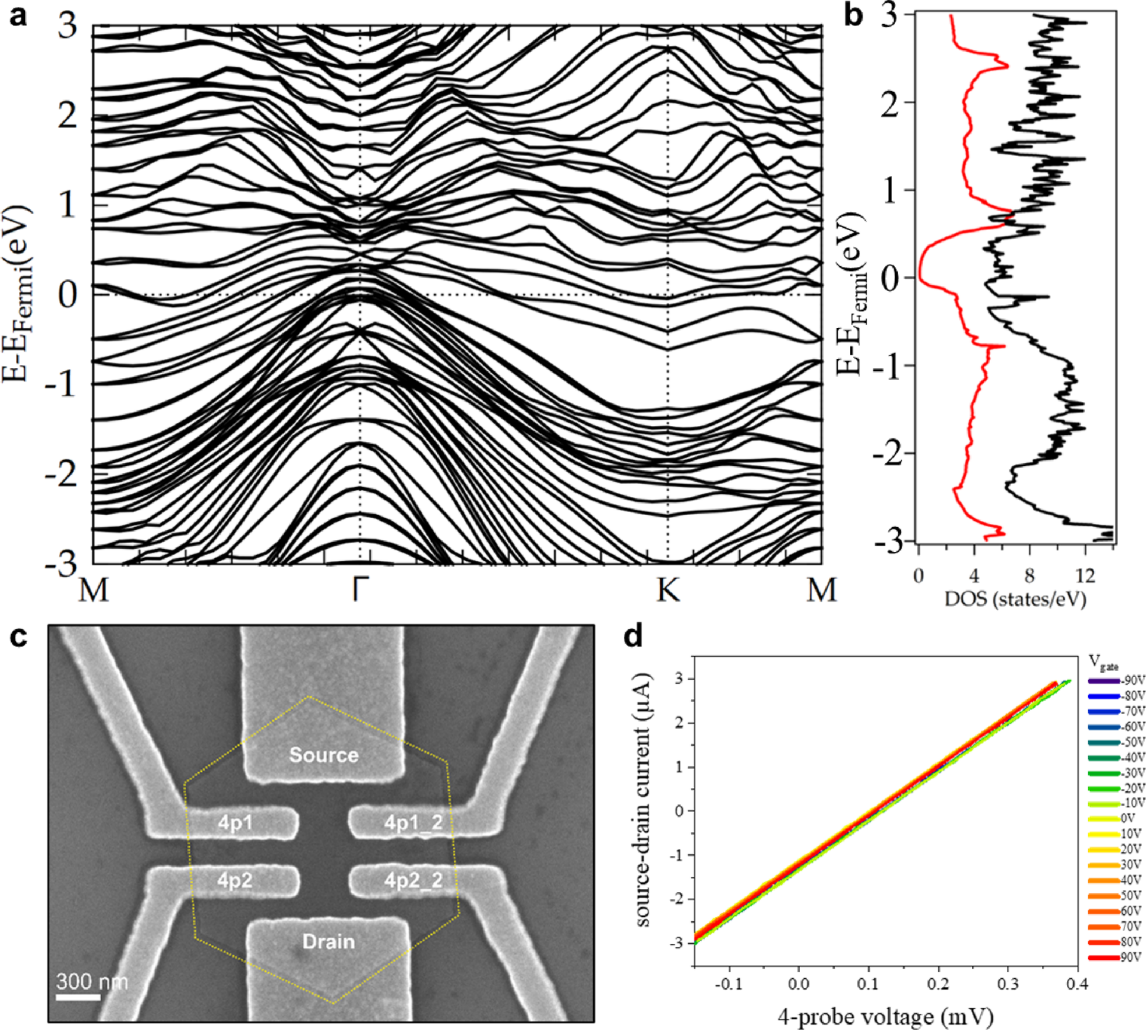
Electronic properties and transport measurements of the hybrid bismuth/bismuthene heterostructure. (a) Electronic band structure of the bismuth/bismuthene hybrid, obtained by *ab initio* calculations. (b) Projected density of states for the hybrid material (black) and β-bismuth (red). Newly formed states around the Fermi energy are generated for the hybrid material. (c) SEM image of contacted individual crystallite for transport measurements. (d) *I*–*V* curves showing a linear behavior for different gate voltages ranging from −90 to +90 V for room temperature transport measurement.

The clarification of the thiol-stabilized hybrid structure now leads to the use of the structural model to further explore the expected electronic peculiarities of the hybrid material. The calculated band structure in Figure 4a reveals a rich variety of newly established bands crossing the Fermi level (see also Supplementary Figure 18 for the band structure of β-bismuth), emerging from the atomic arrangement of the hybrid structure and expecting the surface to behave as a metal. This is also represented by the projected density of states (DOS), as shown in Figure 4b, where a variety of newly formed states appears around the Fermi energy. The surface spectrum along M-Γ-K was rationalized by creating a half-layer terminated Bi structure (Supplementary Figure 25), which reveals six surface states in the vicinity of K (Supplementary Figure 28). The postulated metallic nature of the materials’ surface fits to room temperature transport measurements of an individual flake of the hybrid material, shown in Figure 4c (several devices have been prepared showing reproducible data). During the measurements, the samples were stored at room temperature and in high-vacuum conditions (<10^–4^ mbar). The two-probe measurements of each contact pair show ohmic behaviors and are very stable up to 10 mV without any hysteresis (see Supplementary Figure 20). Although the contact resistance is still included in the measurements, the current is already in the μA-regime and the mean two-probe sheet resistance between source and drain contacts is 4.1 kΩ. Four-probe measurements yield that the mean four-probe resistance is only ∼90 Ω (square resistivity ρ_□_ = 45 Ω), without any non-linearity up to 10 mV. For further investigations, different voltages were applied to the back gate of the sample, ranging from −90 V to +90 V. As can be seen in Figure 4d, the four-probe IV curves do not change while varying the gate voltage. This strict and reproducible linearity of the IV-characteristics displays a consistent metallic behavior (or a very low bandgap semiconductor behavior): no gate response, no Schottky-non-linearity at the contacts. As the measurements were carried out at room temperature and not under low temperature conditions, the metallic transport behavior might be also interpreted as transport in a low-bandgap semiconductor and the interaction of the electron beam during lithographic structuring of the electrodes might locally alter the composition of the surface layer and lead to a desorption of sulfur functionalization; nevertheless, based on the microscopic and catalytic data, it is safe to assume the actual metallic behavior of the surfaces of the investigated hybrid materials.

In the band structure of pure Bi, presented in Supplementary Figure 18, no states are crossing the Fermi level as extracted from our DFT calculations. The novel obtained functionalized hybrid on the other hand shows a change in the calculated band structure (Figure 4a) where new states appear crossing the Fermi level and thus obtaining a highly conductive system as proved by the density of states simulation. From our molecular interfacial engineering approach, we therefore now have a material at hand that constitutes a coherent sandwich, consisting of an almost insulating Bi core and terminating layers of covalently functionalized bismuthene. The resulting chemical asymmetry due to the attached thiol groups can be expected to generate intrinsic dipolarity^40,41^ and might thus lead to the direct generation of quantum spin hall (QSH) effects, as proposed for other functionalized 2D Bi arrangements. In line with earlier work of bismuthene on SiC, the correlation of the 30° rotated superstructure with respect to the substrate and the enlarged lattice constant also here might lead to dramatic changes in the electronic behavior of the material.^17^ Other bismuth-based materials have been shown to offer a rich variety of physical phenomena,^42−44^ and we foresee that our sandwiched material will become a new promising member of this materials class. Furthermore, this synthetic approach can be extended to other members of the pnictogen family.

## Conclusions

In conclusion, we presented the colloidal synthesis of a high-quality 2D bismuth hybrid material by a purely wet chemical approach. The presented material combines the possibilities of upscaling, shows unexpected plasmonic modes, and behaves as a coherent semi-metal/metal hybrid structure. The intrinsic functionalization leads to the stabilization of the bismuth interface structure while enabling a remarkable protection of the surface against bismuth oxide formation, as demonstrated by several spectroscopic and catalytic experiments. This bismuth/bismuthene hybrid material will pave the way for a deeper exploration of novel physical properties and will allow the use of this material in organic catalysis, biomedicine, plasmonics, or energy storage and conversion.

## Methods

### Colloidal Synthesis of Bismuth Hybrids

A 100 mM stock solution of bismuth neodecanoate (Bi(OCOC(CH_3_)_2_(CH_2_)_5_CH_3_)_3_, Sigma-Aldrich) in 1-octadecene (C_18_H_36_, Sigma-Aldrich, technical grade, solvent) was prepared by weighting 3.614 g (5 mmol) of bismuth neodecanoate in a 50 mL measuring flask that was filled up with 1-octadecene. For the colloidal synthesis, 1.25 mL ([Bi^III+^] = 5 mM, 125 μmol, 1 equiv) of the stock solution was mixed with 22.22 mL of 1-octadecene and 1.23 mL of oleylamine (CH_3_(CH_2_)_7_CH=CH(CH_2_)_7_CH_2_NH_2_, Sigma-Aldrich, >98%, 3.75 mmol, 30 equiv) into a 100 mL two-neck flask. The flask was evacuated and heated in an oil bath (200 ° C) under stirring (400 rpm) with continuous illumination by a commercial white light LED (Philips). After degassing for 13 min, the atmosphere was changed to Ar and the reaction mixture was left to stir for another 2 min. Through a septum, 0.3 mL of 1-dodecanethiol (CH_3_(CH_2_)_11_SH, Sigma-Aldrich, >98%, 1.25 mmol, 10 equiv) was injected and the mixture was stirred until a clear color change from yellow to black was noticed. The reaction time is dependent on the reaction temperature and light intensity (see Supplementary Figure 1). The obtained product was then quenched in an ice bath and subjected to centrifugation (10 min, 10 krpm) and redispersion/washing cycles (three times) in chloroform inside an Ar-filled inert gas glovebox.

Specimens for further characterization were prepared from the suspension in chloroform (TEM, SEM, AFM, Raman spectroscopy, and XPS) or using the dried solid (PXRD).

All materials were used as received without any further purification.

### Microscopy

Transmission electron microscopy was carried out using a Titan Themis 300 (300 kV, mono-STEM-EELS, EDX), a Titan^3^ G2 (300 kV, ac-HRTEM), a FEI Osiris (200 kV, STEM-EDX), and a JEOL ARM 200F (200 kV, HRSTEM, aberration-corrected STEM-EELS).

Plasmon maps were recorded using a semi convergence angle of 15.7 mrad and a collection semi angle of 37.5 mrad in a Gatan Quantum spectrometer with an energy resolution of 0.01 eV/channel. The microscope was operated in STEM mode using a camera length of 29.5 mm.

FIB lamella preparation was done in a Helios G4 system (see Supplementary Figure 9).

AFM measurements were conducted using a Nanoscope IVa Multimode Scanning Probe Microscope (Bruker, former Veeco) in tapping mode.

### Physical Characterization

XRD was carried out with a PANalytical Empyrean X-ray platform using Cu Kα radiation in θ–2θ geometry and an angular range from 2 to 70° in 0.02° steps. The sample was kept in a glass capillary under ambient conditions.

Raman spectroscopy characterization was carried out using a Horiba LabRam HR Evolution spectrometer, employing a HeNe laser (632.8 nm). The laser was focused using a 100× objective (0.8 NA), thus leading to a laser spot with a diameter of *ca*. 1 μm. An EMCCD camera was employed to collect the backscattered light that was dispersed by an 1800 grooves per mm grating providing a spectral resolution of ∼1 cm^–1^. The corresponding Raman spectra were then constructed by processing the data using Lab Spec 6 software.

For XPS measurements, a TFS K-Alpha X-ray Photoelectron Spectrometer (K-Alpha compact XPS) was employed. The powdered sample was glued to a sample holder using carbon tape. The primary radiation source was monochromatized Al Kα (1486.6 eV) using a spot size of either 200 or 400 μm. The base pressure for the measurement was 4 × 10^–9^ mbar. For survey spectra, the analyzer energy and spectral resolution were set to 150 and 1 eV, respectively. High-resolution spectra were acquired using an analyzer energy of 50 eV and a spectral resolution of 0.1 eV.

Etching of the surface was carried out with an Ar ion beam (2 kV, 7.8 μA) for 30 s, effectively removing the functionalized surface layer.

### Plasmon Simulation

Numerical simulations of EEL spectra have been carried out using the MNPBEM package for Matlab, developed by U. Hohenester and A. Trügler at the University of Graz.^45−47^ We consider hexagonal bismuth flakes with round tips embedded in vacuum with sides ranging from 250 to 500 nm (*i.e*., edge-to-edge diameters from 0.5 to 1 μm) and thickness up to 12 nm. The surface of the flake is discretized in a fine triangular mesh with more than 10,000 faces for the larger flakes. The structure is mapped by a sub-nanometer electron beam and the resulting polarizability calculated using retarded green functions to observe the dependence of the EEL spectrum on the position. This way we can foresee the energies expected for localized surface plasmon resonances and reproduce their modal distribution. In particular, the spectra compared with the experimental data of Figure 1 and Supplementary Figure 3 are obtained, considering a hexagonal flake of 920 nm diameter and a thickness of 10 nm. We used the dielectric function from Toudert *et al*.^30,48,49^

### Electronic Structure Calculations

We performed density functional theory (DFT) calculations within the generalized gradient approximation (GGA) using the Quantum ESPRESSO package.^50^ The Perdew–Burke–Ernzerhof (PBE)^51^ functional was used to describe the exchange–correlation energy. We employed fully relativistic ultrasoft pseudopotentials to account for spin–orbit coupling effects. The electronic wave functions were expanded with well-converged kinetic energy cutoffs of 80 and 480 Ry for the wave functions and charge density, respectively. The Brillouin zone was sampled by a fine Γ-centered 8 × 8 × 8 *k*-point Monkhorst–Pack^52^ mesh for the case of the bulk system and 10 × 10 × 2 for the slab calculations, where a vacuum of 18 Å was added in the *c* direction to avoid interactions due to periodic boundary conditions. To account for van der Waals interactions between the layers, we applied semi-empirical Grimme-D2 dispersion corrections.^53^ All the structures were fully optimized using the Broyden–Fletcher–Goldfarb–Shanno (BFGS) algorithm^54^ until the forces on each atom were smaller than 1 × 10^–3^ Ry/au and the energy difference between two consecutive relaxation steps was less than 1 × 10^–4^ Ry.

We first computed a pristine bismuth bulk system containing six atoms in the unit cell that belong to three layers arranged in an ABC stacking. After the structural optimization, the computed lattice parameters are *a* = *b* = 4.47 Å and *c* = 11.99 Å. The electronic band structure reveals a semimetallic behavior, as one can observe in Supplementary Figure 18, due to a band crossing the Fermi level along the path Γ-A (*z* direction in real space), which results in a low density of states at the Fermi level. The orbital resolved density of states of the bulk structure indicates that s and p orbitals are the ones that describe the bands over a wide range of energies around the Fermi energy (Supplementary Figure 21), while the inner *d* orbitals are placed at lower energies.

To consider surface state effects, we computed a slab system formed by six layers of pure buckled bismuth containing 12 atoms, thus having an ABCABC stacking along the *z*-direction. A vacuum of 18 Å in the *z* direction was added. After structural optimization, the lattice parameter was reduced by a 3%, *i.e*., *a* = 4.34 Å, due to the change of screening at the surface. The distance between the layers is ∼3.73 Å.

We can observe two surface bands crossing the Fermi level along the Γ-M path (Supplementary Figure 22), which have been reported to play an important role in the electronic transport properties of ultrathin Bi films.^55^

To unveil the structural and electronic properties of the hybrid structure, we constructed a model formed by six layers of pristine bismuth arranged in an ABCABC stacking along the *z*-direction, capped by an additional half layer of bismuth that is covalently bonded to a thiol chain via a Bi–S chemical bond. For simplicity reasons, we employed a hexanethiol chain in the calculation formed by one atom of S, 6C, and 13H with a total length of 8.71 Å, thus reducing the computational cost. We investigated the possibility of having such a chemical functionalization due to the evidence of the presence of S bonded to Bi in the surface of the material (see the EDX and XPS spectrum in Figure 2b,c, respectively) and the use of dodecanethiol molecules as a reducing agent in the chemical reaction (see more details in Figure 1a). This hypothesis allowed us to reach the minimum potential energy and stabilize the proposed hybrid structure containing a half layer of bismuth on the surface. The calculated lattice parameter *a* = 4.35 Å is very close to the one calculated for the slab of pristine Bi. The fully optimized atomic positions and cell parameters are reported in Supplementary Table 2. In this structure, we could observe that the bismuth layers that are closer to the surface tend to get more compacted, resulting in 3.17 Å distance between the two layers that are next to the functionalized surface in comparison to the bulk equidistant separation of 3.73 Å. As one moves toward the inner layers of the bismuth/bismuthene material, our calculations reach a constant distance of 3.71 Å, which is very similar to the bulk one, showing that the effects of functionalization are negligible there.

Regarding the electronic structure, the calculations evidence a metallic behavior coming from the presence of half-filled bands crossing the Fermi energy in all the Μ-Γ-K plane. The band structure is plotted in Figure 4a. These result in different channels for electric charge conductivity. The strong metallic character of the hybrid system is also observable in the projected DOS presented in Figure 4b. The orbital resolved DOS (Supplementary Figure 23) indicates that most of the metallic character is caused by the p orbitals of bismuth. This suggests that the thiol chains are key to stabilize the hybrid structure, but the enhanced surface conductivity comes from the bismuth contribution.

### Tight-Binding Model

To further understand the contribution of having a half-layer terminated Bi system, we derived a tight-binding Hamiltonian based on maximally localized Wannier functions (MLWFs)^56−58^ using the s and p orbitals of Bi as a basis set in the Wannier90 code^59^ (Supplementary Figure 24). The selection of these projectors is supported by the orbital resolved DOS shown in Supplementary Figure 23. To rationalize the effect of the surface states in the electronic structure, we designed two model systems of Bi. The first one (model A) considers a hexagonal unit cell of bulk Bi formed by three layers with an ABC stacking, thus being terminated by a buckled bismuth layer. On the other hand, for the model B, we considered the same unit cell but finishing both top and bottom surfaces in half layer of 2D bismuth (see Supplementary Figure 25). The derived Wannier tight-binding Hamiltonian was introduced as an input in the WannierTools package^60^ that permitted us to replicate the unit cells proposed for models A and B by 10 in the *z* direction adding a vacuum to simulate a (111) surface. This led to the determination of the Fermi surface and surface spectral functions for semi-infinite Bi(111) surfaces using the surface Green’s function method of a system formed by (i) 30 layers of β-bismuth (model A) and (ii) 29 layers of β-bismuth with the two surfaces of bismuth (model B).

The simulation of the ARPES, Fermi surface, and spin texture of model B are shown in Supplementary Figure 19. Results of model A are shown in Supplementary Figures 26 and Figure 27. Our calculations of the surface states of a slab system based on β-bismuth are in excellent agreement with photoemission experiments^61^ and previous ARPES calculations.^10^ Comparing the ARPES simulation of β-bismuth (Supplementary Figure 26) with the structure terminated in the half layer of bismuth (Supplementary Figure 19), we can observe in the latter the formation of four bands along the Γ-K-M direction around the Fermi energy due to the larger delocalization of the charge density on the surface of the material as a consequence of the loss of the bonding with the removed Bi atoms. This suggests a metallic behavior in agreement with the linearity of the surface transport measurements, which seems to have its origin in the surface Bi p orbitals.

### TEM Data Simulation

The optimized structures extracted from the DFT calculations were used as input datasets for HRTEM and diffraction simulation using the Computem package from Earl J. Kirkland. For HRTEM simulation, the parameters were as follows: 300 kV, objective aperture: 20 mrad, defocus spread: 4.5 nm, C_s_: 5 μm and varying defocus values.

### Catalytic Tests

All chemicals were of reagent grade quality, purchased from commercial sources, and used as received. Reactions were performed in 10 mL round-bottom flasks equipped with a magnetic stirrer, closed with a rubber septum for sampling, and placed in oil baths at the required reaction temperature. Glassware was previously dried at 175 °C in an oven before use. Gas chromatographic analyses were performed in an instrument equipped with a 25 m capillary column of 5% phenylmethylsilicone. *n*-Dodecane was used as an external standard. GC/MS analyses were performed on a spectrometer equipped with the same column as the GC and operated under the same conditions. All the organic products obtained were characterized by GC–MS, after comparison with commercial samples.

#### Representative Procedure for the Decarboxylative Oxidation Reaction

4-Bromo-dl-mandelic acid (2 mmol) was dissolved in DMSO (5 mL) in the presence of Bi(0) powder (0.2 mmol) and 3 mmol of AcOH (50% aqueous solution). The mixture was stirred at 125 °C under an atmosphere of oxygen (1 bar, balloon) until complete consumption of the starting material, which was assessed by GC and GC–MS with *n*-dodecane as an external standard. 84% of 4-bromo benzoic acid was obtained.

#### Representative Procedure for the Friedel–Crafts Acylation Reaction

A round-bottom flask was charged with Bi_2_O_3_ (0.233 g, 0.5 mmol) and [emim][NTf_2_] (0.391 g, 1 mmol). After drying under vacuum for 1 h at 40 °C, with stirring and flushing several times with nitrogen gas, toluene (2.14 mL, 20 mmol) was introduced. The mixture was heated at 150 °C, and benzoyl chloride (1.11 mL, 10 mmol) was added. Upon completion of the reaction, the mixture was analyzed by GC–MS with *n*-dodecane as an external standard to obtain 80% yield of methylated benzophenone product, in a composition *o*:*m*:*p* 1:5:18.

### Transport Measurements

To contact the synthesized bismuth hybrids, we chose a SiO_2_/Si wafers as a substrate. The highly doped silicon can act as a gate electrode, which is contacted from the backside. A position marker grid consisting of titanium-gold crosses was evaporated on the chip. Subsequently, the bismuth flakes were transferred on the chip via drop-casting after their synthesis following the previously described procedure. Six electrical contacts to the bismuth flakes in FET configuration were patterned by e-beam lithography utilizing a SUPRA Zeiss SEM (10 keV) and a lift-off technique. As contact materials, titanium with a layer thickness of 5 nm and gold on top with 40 nm thickness were applied.

Measurements were carried out at room temperature and under high vacuum conditions in different devices, showing reproducible results.

## Supporting Information Available

Video of redox reaction after addition of DDT (AVI)
Focus series (ac-HAADF-STEM) of bismuth/bismuthene hybrid obtained at 200 kV (AVI)
Additional synthesis of materials, characterization techniques, lamella preparation, microscopy and transport characterization, theoretical calculations, and additional catalytic results (PDF)

## Supplementary Material

ja2c13036_si_001.avi

ja2c13036_si_002.avi
