# Supplementary material for: Hexagonal Hybrid Bismuthene by Molecular Interface Engineering
Source: J Am Chem Soc. 2023 Jun 1;145(23):12487–98. doi: 10.1021/jacs.2c13036 (PMC10273315; doi:10.1021/jacs.2c13036)
Supplement: Supplementary file 3 — ja2c13036_si_003.pdf [file ja2c13036_si_003.pdf]

Supplementary information for

Hexagonal hybrid bismuthene by molecular interface engineering

Christian Dolle[1, †,‡], Víctor Oestreicher[1, ‡], Alberto M. Ruiz[1], Malte Kohring[2], Francisco Garnes-Portolés[3], Mingjian Wu[4], Gabriel Sánchez-Santolino[5], Alvaro Seijas-Da Silva[1], Marta Alcaraz[1], Yolita M. Eggeler[6], Erdmann Spiecker[4], Josep Canet-Ferrer[1], Antonio Leyva-Pérez[3], Heiko B. Weber[2], María Varela[5], José J. Baldoví[1], Gonzalo Abellán[1]*

[1]Instituto de Ciencia Molecular (ICMol); Universidad de Valencia, Catedrático José Beltrán Martínez nº 2, 46980 Paterna, Spain.

[2]Lehrstuhl für Angewandte Physik; Friedrich-Alexander Universität Erlangen-Nürnberg (FAU), Staudtstr. 7, 91058 Erlangen, Germany.

[3]Instituto de Tecnología Química (UPV–CSIC), Universitat Politècnica de València–Consejo Superior de Investigaciones Científicas, Avda. de los Naranjos s/n, 46022 Valencia, Spain.

[4]Institute of Micro- and Nanostructure Research (IMN) & Center for Nanoanalysis and Electron Microscopy (CENEM); Interdisciplinary Center for Nanostructured Films (IZNF), Friedrich-Alexander Universität Erlangen-Nürnberg (FAU), Cauerstraße 3, 91058 Erlangen, Germany.

[5]Instituto Pluridisciplinar & Departamento de Física de Materiales; Universidad Complutense de Madrid (UCM), Plaza de Ciencias 1, Ciudad Universitaria, 28040 Madrid, Spain.

[6]Laboratory for Electron Microscopy (LEM), Microscopy of Nanoscale Structures & Mechanisms (MNM); Karlsruhe Institute of Technology (KIT), Wolfgang-Gaede Str. 1a, 76131 Karlsruhe, Germany.

*Corresponding author. Gonzalo Abellán: Email: Gonzalo.abellan@uv.es

[†]Present address: Laboratory for Electron Microscopy (LEM), Microscopy of Nanoscale Structures & Mechanisms (MNM); Karlsruhe Institute of Technology (KIT), Wolfgang-Gaede Str. 1a, 76131 Karlsruhe, Germany

[‡]These authors contributed equally to this work

This PDF file contains:

Supplementary Tables 1 to 3
Supplementary Figures 1 to 28
Caption for Supplementary Movies SM1 and SM2

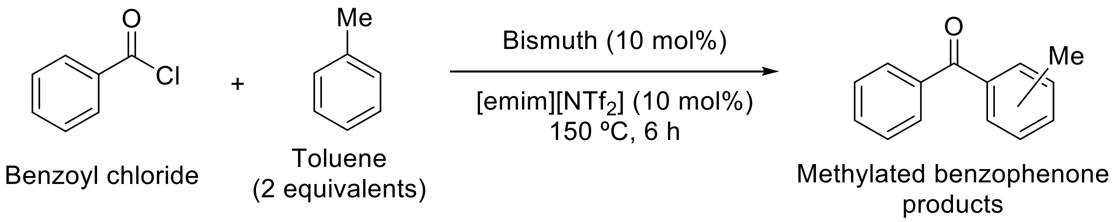

Supplementary Table 1. Catalytic results for the Friedel-Crafts coupling of benzoyl chloride with toluene catalyzed by 10 mol% of different bismuth species, under the above indicated reaction conditions. Gas-chromatography yields. Selectivity towards the Friedel-Crafts products is 100% (no other products found).

Entry	Catalyst	Time in air (months)	Yield (%)
1	Bi^0	-	0
2	Bi_2O_3	-	80
3	Unprotected bismuth particles	0	0
4		0.5	35
5		2	67
6		3	69
**7**	**2D sandwiched bismuth/ bismuthene hybrid**	**0**	**0**
**8**		**3**	**8**
**9**		**6**	**12**

Supplementary Table 2. Atomic positions and lattice parameters of hybrid structure.

ATOMIC POSITIONS (angstrom)

Bi	0.004490107	0.000736497	4.192445235
Bi	2.161686689	1.246862848	1.455270621
Bi	2.233898882	1.291451917	8.499130790
Bi	0.002146351	2.511581184	5.881521447
Bi	-0.012482190	2.504109337	-0.247369313
Bi	0.065290093	0.036000034	10.207718538
Bi	0.065125897	0.105144438	16.810329313
Bi	2.336767032	1.362157377	14.476378647
Bi	2.183676899	1.349628405	20.779068985
Bi	0.174342456	2.654824372	18.648026333
Bi	0.116734567	2.603689647	12.721872864
Bi	0.253395384	0.163402911	22.755334194
Bi	0.079402029	2.604009822	24.774345707
S	-1.441839324	1.629055467	26.676795664
H	-0.300102503	2.02115524	35.391076497
C	-0.330901526	1.817493982	28.136593778
C	-0.957598203	2.012627607	34.509934453
C	-1.134079989	1.885123145	29.429656580
C	-0.153464688	2.005222955	33.210859339
C	-0.232742282	1.947750247	30.662197656
C	-1.030316106	1.984824708	31.961646924
H	-1.608325146	2.899455099	34.566300365
H	-1.791441920	1.003164256	29.512136837
H	-1.601471287	1.121929057	34.577244394
H	-1.797186595	2.765899013	29.410855587
H	-1.688792941	2.871213050	31.956366240
H	-1.697870099	1.106438312	31.997162978
H	0.365910979	0.966866009	28.150332017
H	0.260045372	2.734918397	27.997233428
H	0.504496764	2.889205679	33.173967030
H	0.440376278	1.073186209	30.671053298
H	0.515260429	1.127750902	33.191915667
H	0.419420780	2.835700947	30.600705306

CELL PARAMETERS (angstrom)

4.351984579	0.000000000	0.000000000
-2.175992290	3.768929202	0.000000000
0.000000000	0.000000000	51.799801310

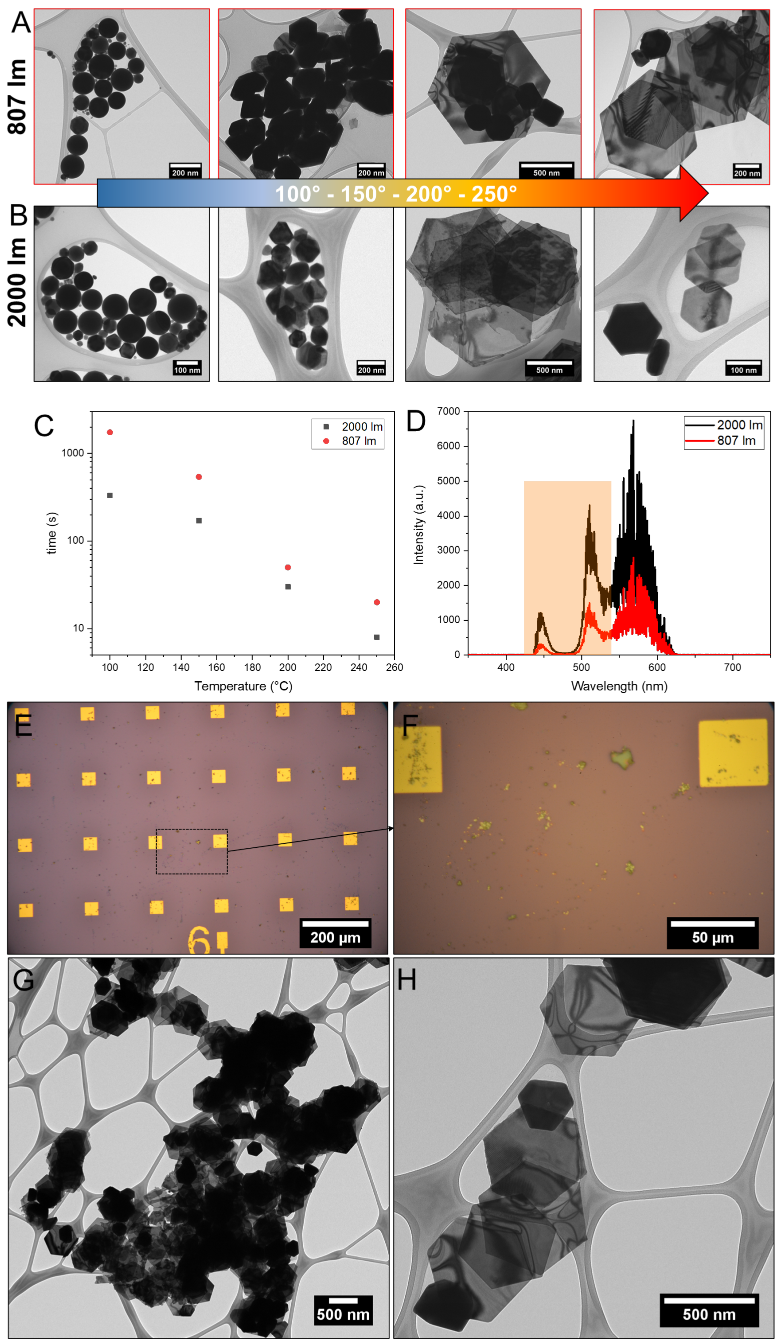

Supplementary Figure 1. Influence of light and temperature on reaction rate and resulting shape and overview images. (**A**) Evolution of particle shape under low-intensity illumination (807 lm, white light LED) in temperature range 100° C–250° C. (**B**) Influence of light intensity on particle shape at high illumination intensity (2k lm). (**C**) Reaction time *vs*. temperature plot. Here, the end of the reaction was assumed as soon as the reaction mixture turned into a black suspension (also see Supplementary Movie SM1). (**D**) Spectral fingerprint of employed white light LEDs. Reaction proceeds upon illumination in indicated wavelength range (blue light). (**E-F**) Optical micrographs and (**G-H**) TEM images showing the prevalence of large (around 1 µm diameter) and thin (pinkish contrast in optical micrograph) particles.

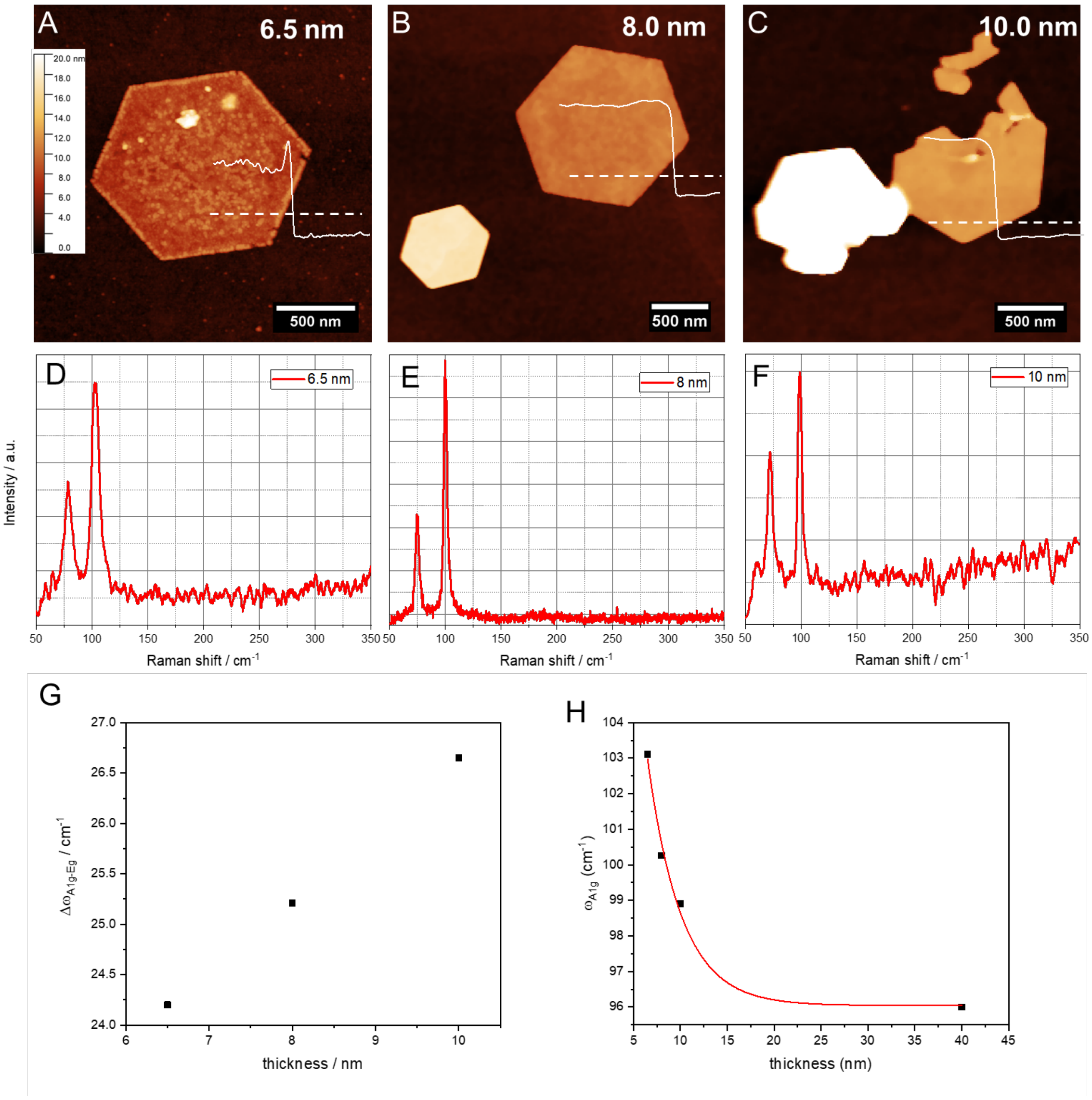

Supplementary Figure 2. Thickness dependent Raman fingerprints. (**A**)-(**C**) AFM data of hybrid materials with different thicknesses as indicated and (**D**)-(**F**) corresponding Raman spectra acquired with HeNe laser. (**G**) Separation of A_{1g} and E_g in wavenumber plotted *vs.* thickness. A clear trend towards smaller shifts for thinner crystallites can be noticed. (**H**) Dependency of A_{1g} shift *vs.* thickness. Upon increasing thickness, the Raman shift for A_{1g} approaches 96 cm^{-1}.

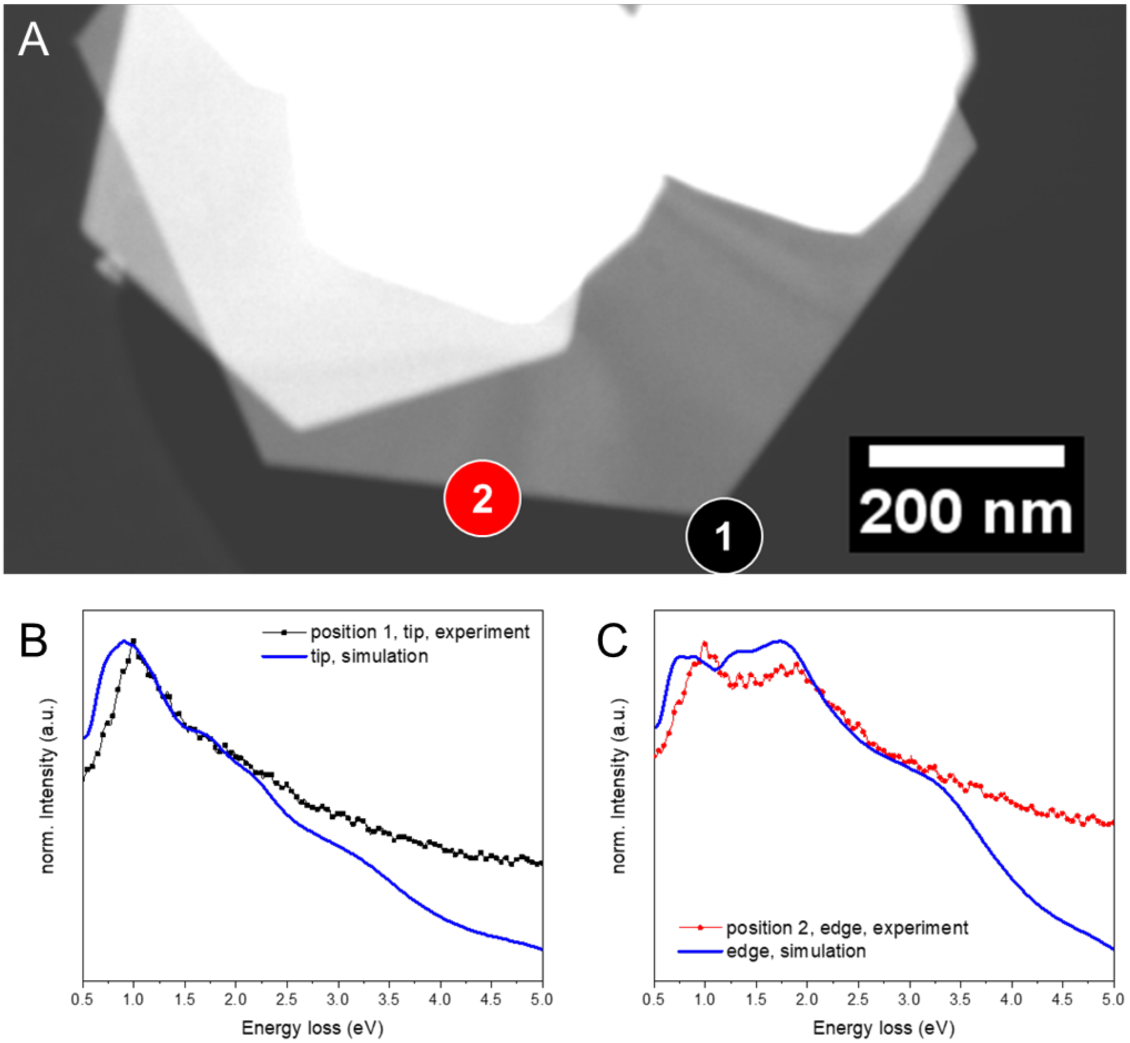

Supplementary Figure 3. Comparison of localized surface plasmon resonances (LSPR) and simulation. (**A**) HAADF STEM data of bismuth/ bismuthene hybrid with two measuring positions indicated. (**B**) and (**C**) normalized experimentally recorded EELS data (after zero loss removal by deconvolution, symbols) with overlayed EELS probability extracted from simulated data of model hexagon (blue line). An excellent match between experiment and simulation for the peak energies can be stated.

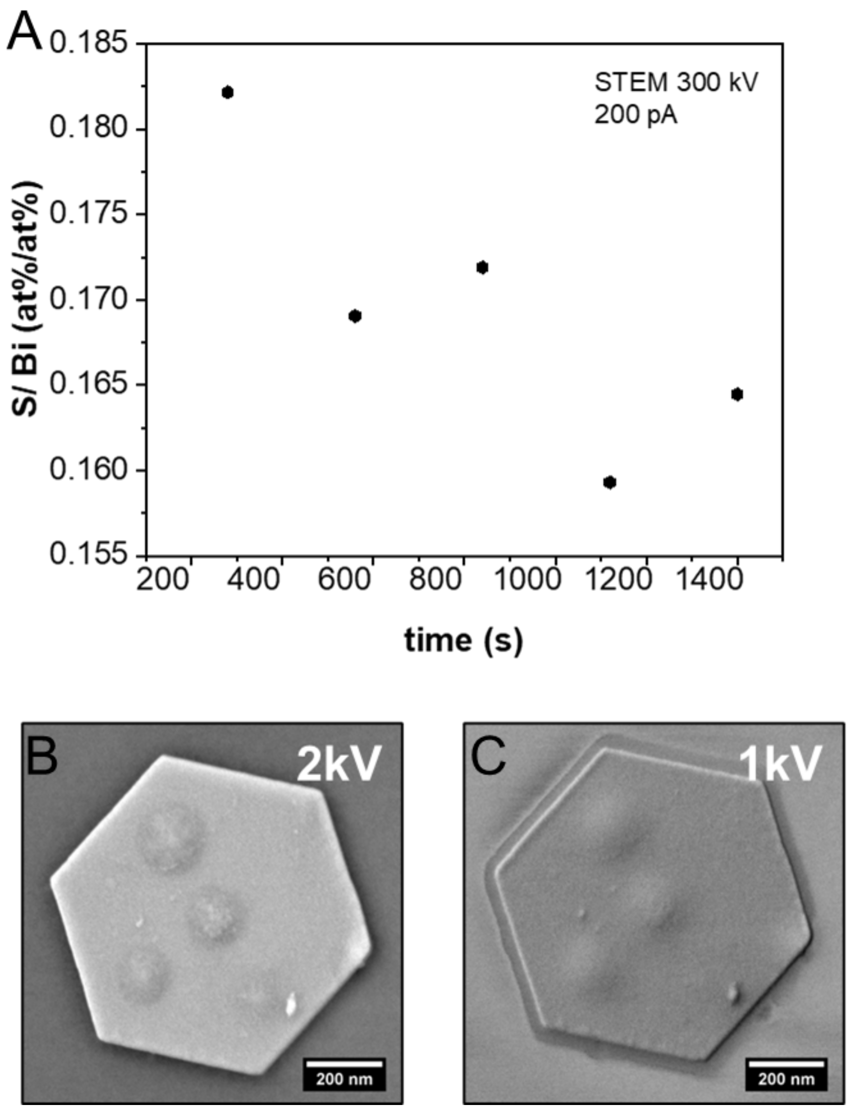

Supplementary Figure 4. Electron beam influence on hybrid material. (**A**) Relative atomic percentage of sulfur/ bismuth, extracted from EDS mapping (see Fig. 2). The data was evaluated by summarizing 20 frames (14 s frametime each) at 5 timesteps during the experiment. A subtle decrease of sulfur over time can be noticed, giving an indication for electron beam induced defunctionalization. (**B**) SEM image of individual crystallite, showing round contrast features after one slow scan image. (**C**) Same crystallite after several slow scan images showing a blister formation under low energy conditions with topography sensitivity. Electron beam induced defunctionalization hereby leads to local strain release and blister formation by surface decoupling.

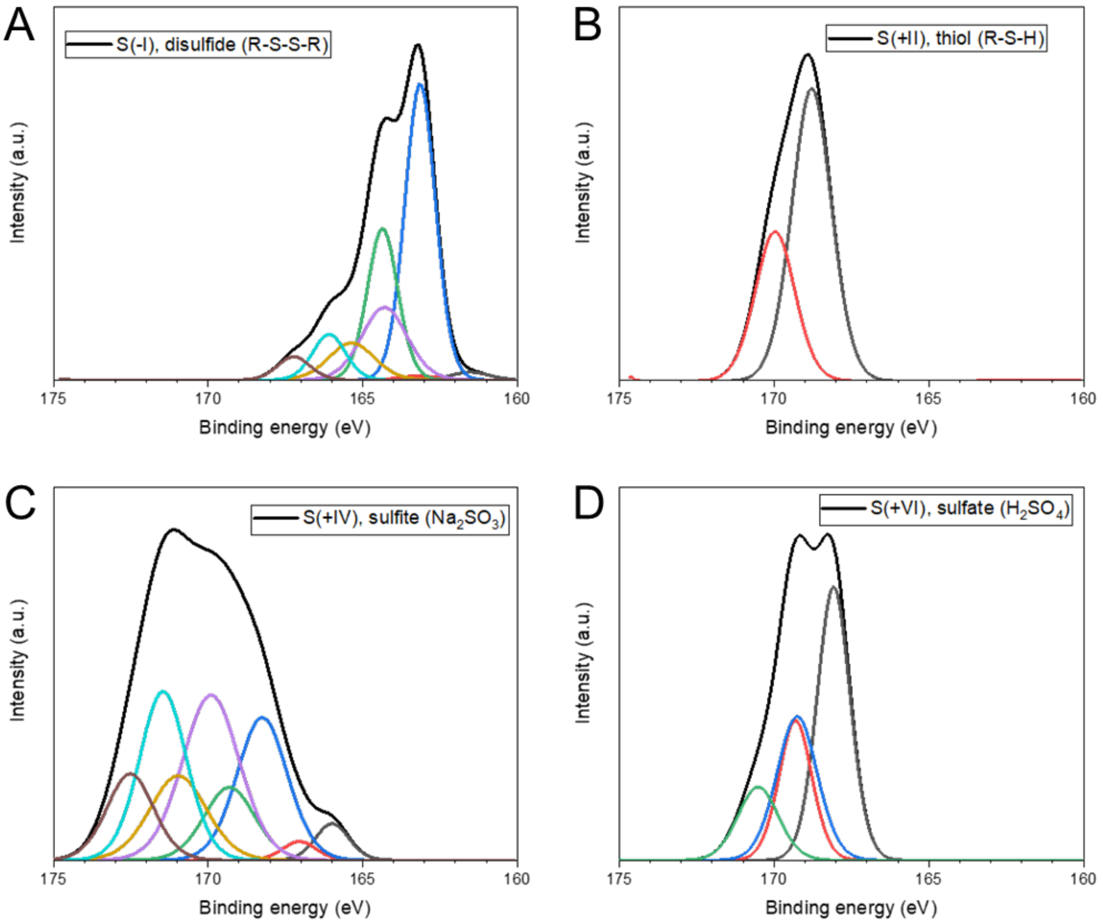

Supplementary Figure 5. Comparison of XPS data for different oxidation states of sulfur S 2p energy. (**A**) S^{-I}, (**B**) S^{+II}, (**C**) S^{+IV} and (**D**) S^{+VI}.

A

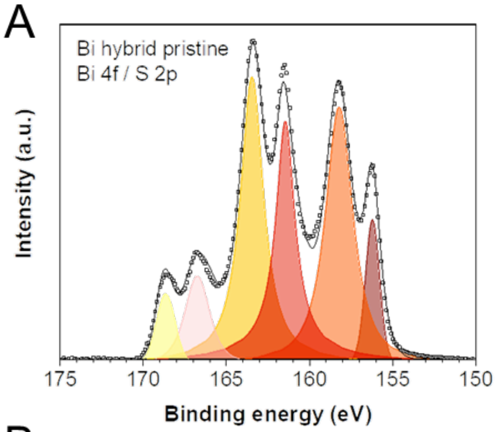

Binding energy (eV)	Orbitals	Bismuth type
156.2	Bi $4f_{7/2}$	Bi^0
158.2		Bi^{III}
161.4	Bi $4f_{5/2}$	Bi^0
163.5		Bi^{III}
166.7	S $2p_{3/2}$	
168.6	S $2p_{1/2}$	

B

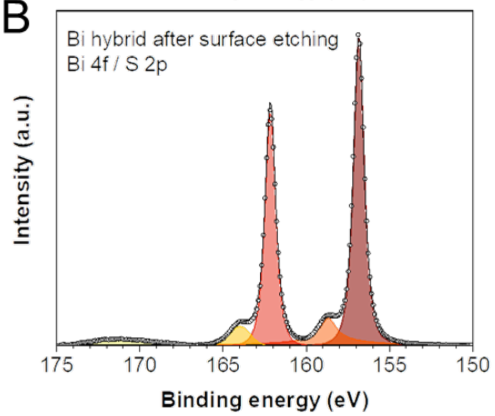

Binding energy (eV)	Orbitals	Bismuth type
156.8	Bi $4f_{7/2}$	Bi^0
158.8		Bi^{III}
162.2	Bi $4f_{5/2}$	Bi^0
163.9		Bi^{III}
171.3	S $2p_{3/2}$	

Supplementary Figure 6. XPS fitting results for bismuth hybrid before and after surface etching. (**A**) Pristine XPS with contributions of Bi^0, Bi^{III} and sulfur, as indicated in table. Sulfur functionalization leads to predominant contribution from Bi^{III} signals due to covalent attachment. (**B**) After surface etching with Ar ions the removal of the functionalized surface leads to a relative increase in metallic Bi. Remaining Bi^{III} contribution originating from not fully sputtered surfaces (see main text).

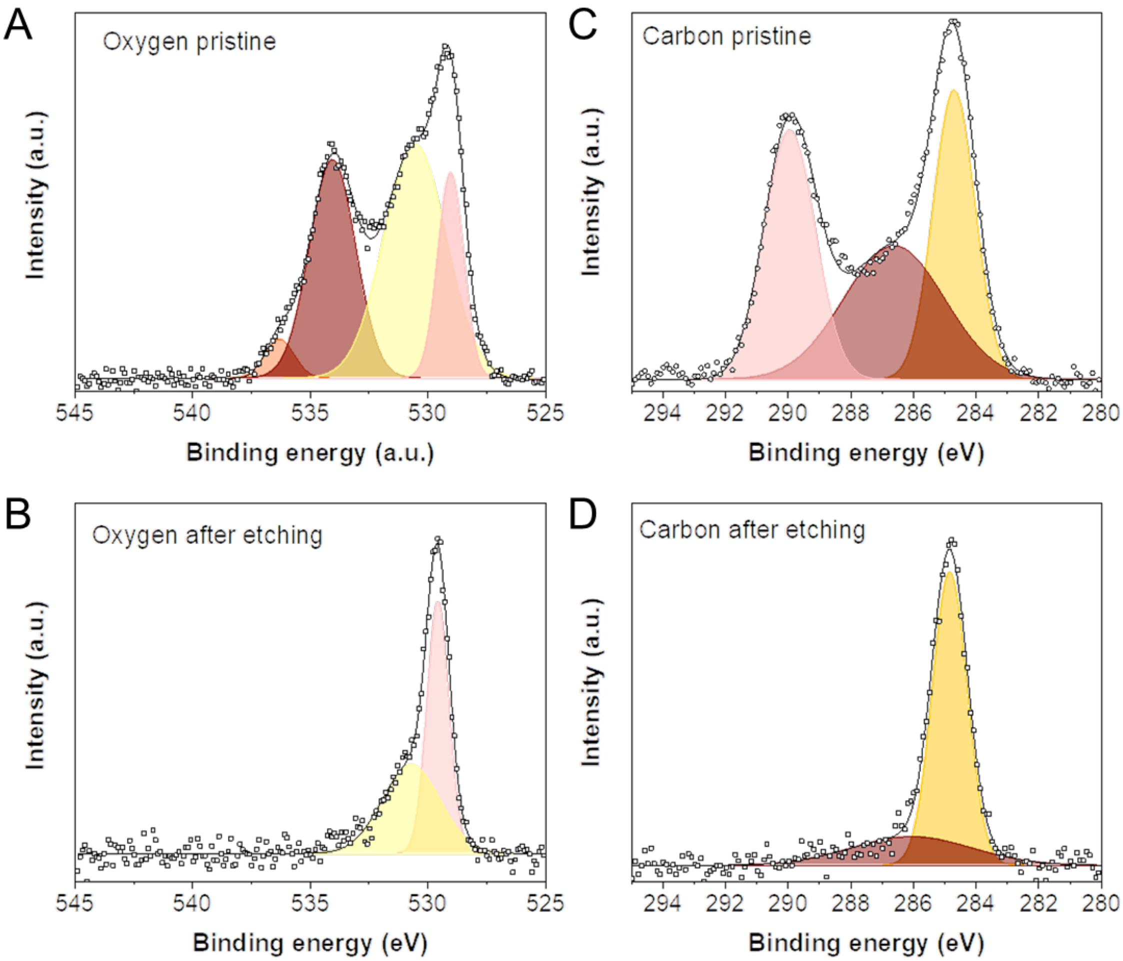

Supplementary Figure 7. High resolution XPS fitting of oxygen and carbon. (**A**-**B**) Fitted oxygen signal before (**A**) and after (**B**) surface sputtering. Contributions >532 eV are removed after etching. (**C**-**D**) Fitted carbon signal before (**C**) and after (**D**) surface etching. Complete removal of contribution >288 eV. Before etching carbon (and oxygen) are contained in sulfur-alkyl functional groups. After removal of surface functionalization carbon and oxygen signal related to sample support.

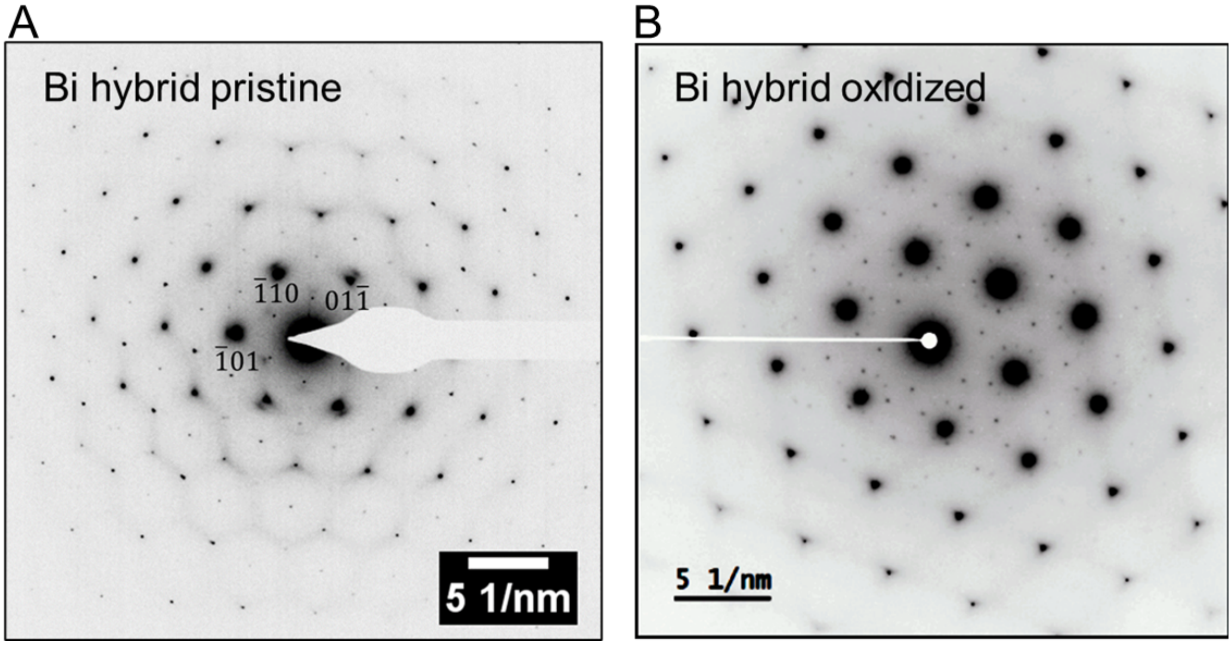

Supplementary Figure 8. Evolution of SAED for oxidation/ aging. (**A**) SAED of individual pristine hybrid material directly after synthesis with Bragg reflections from rhombohedral Bi indicated. Besides the intense Bragg peaks, faint superstructural reflections are visible. (**B**) Bismuth hybrid after storage under ambient conditions for > 6 month. Progressing oxidation leads to appearance of additional reflections from Bi_2O_3.

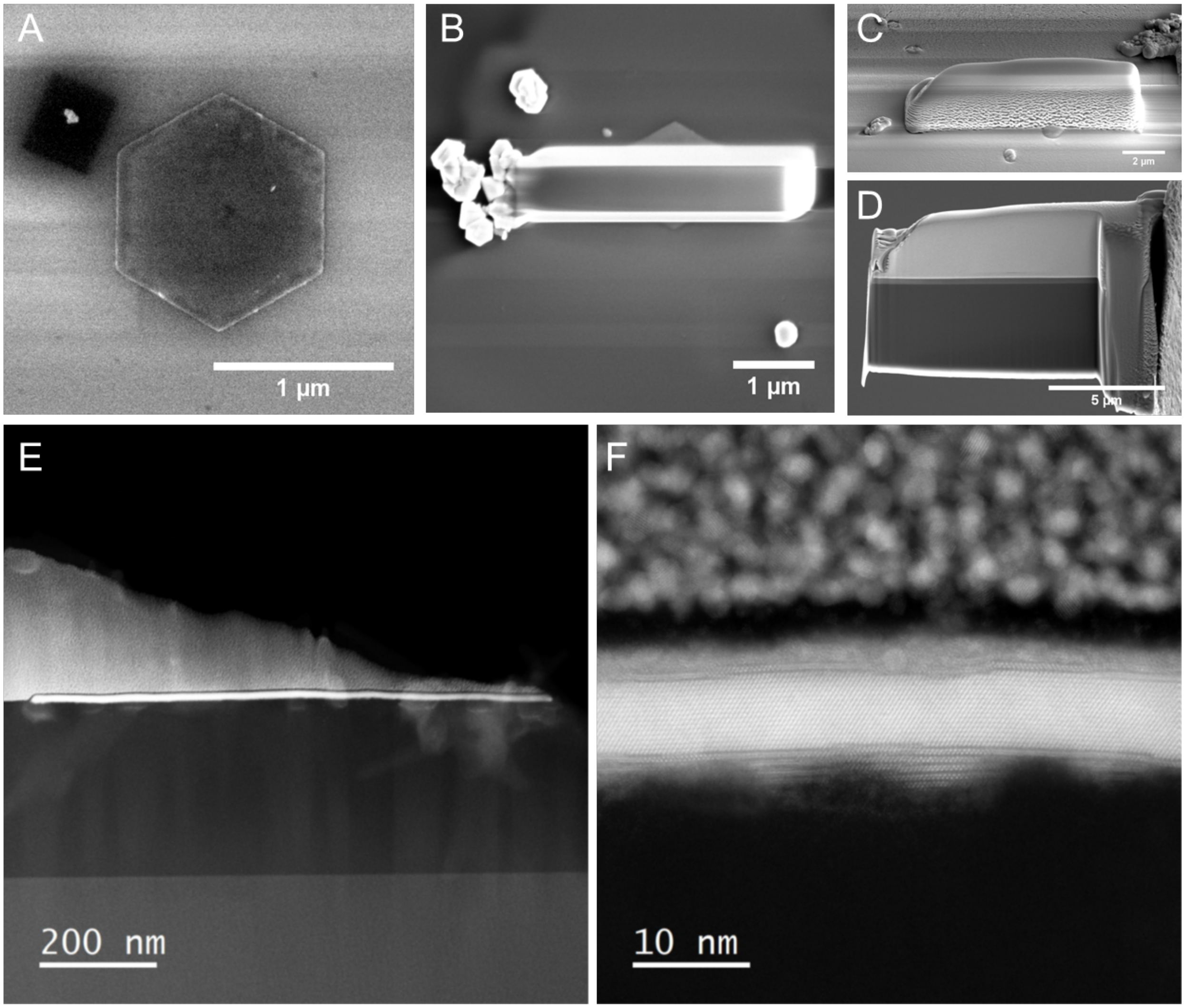

Supplementary Figure 9. Cross section preparation. (**A**) Oriented crystallite, (**B**) and (**C**) after deposition of protective Pt layer. (**D**) Final lamella before thinning to electron transparency. (**E**) Overview HAADF STEM image of lamella. (**F**) HAADF STEM image of the cross section.

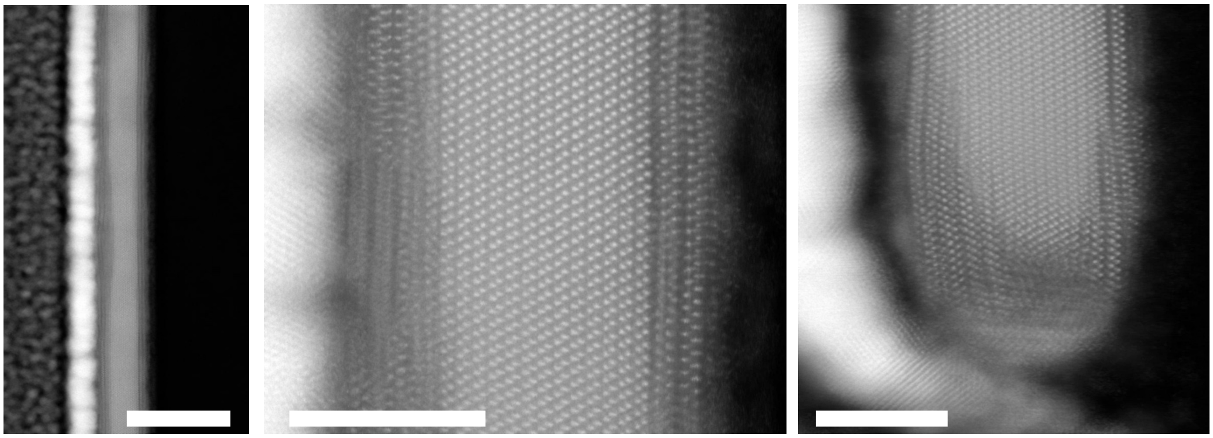

Supplementary Figure 10. **Cross section zoom-in.** High resolution high angle annular dark field (HAADF) scanning transmission electron microscopy cross section images of a Bi flake. The scale bars represent 20 nm (left panel) and 5 nm (middle and right panels). The image on the right corresponds to the edge at the end of the Bi plate. A 1–2 nm thick reconstructed layer is observed both on the top and bottom surfaces of the flake, and also on the edge on the side.

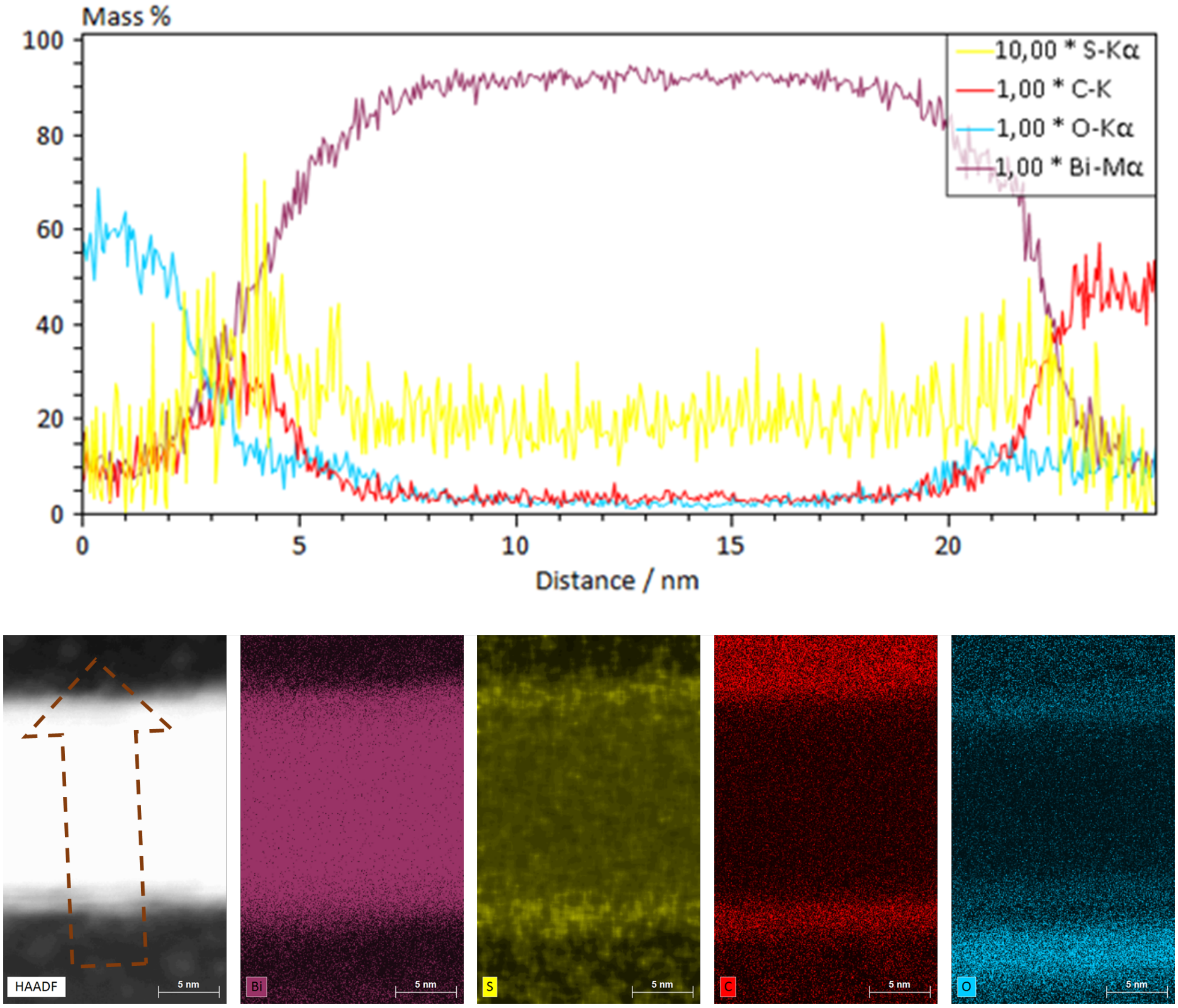

Supplementary Figure 11. Cross sectional STEM-EDX mapping of bismuth hybrid. Line scan evaluation of normalized mass percentage for Bi-M, S-K, C-K and O-K signal. The S-K signal intensity is enhanced by a factor of 10. Line scan extracted at indicated position shown in lower left HAADF-STEM image.

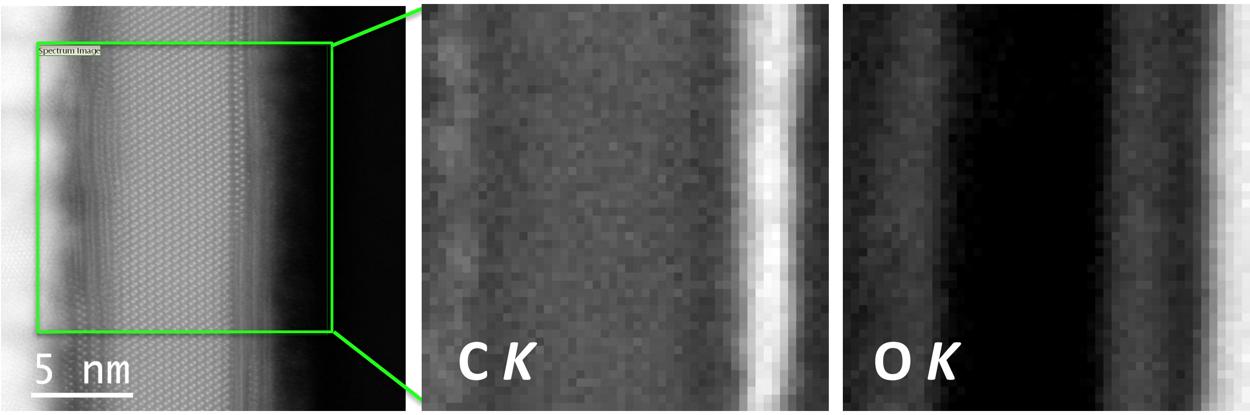

Supplementary Figure 12. STEM-EELS maps of the cross-section Bi flake. The panel on the left shows a high resolution HAADF image. A green square highlights the area where a EEL spectrum image was acquired. The middle and right panels exhibit maps of the C and O signals, respectively. The maps have been produced by integrating 30 eV wide windows under the C K and O K edges, respectively, after background subtraction using a power-law fit. Principal component analysis was used to remove random noise. A slight oxidation of the surface reconstructed layers is detected, along with some C presence related to surface contamination.

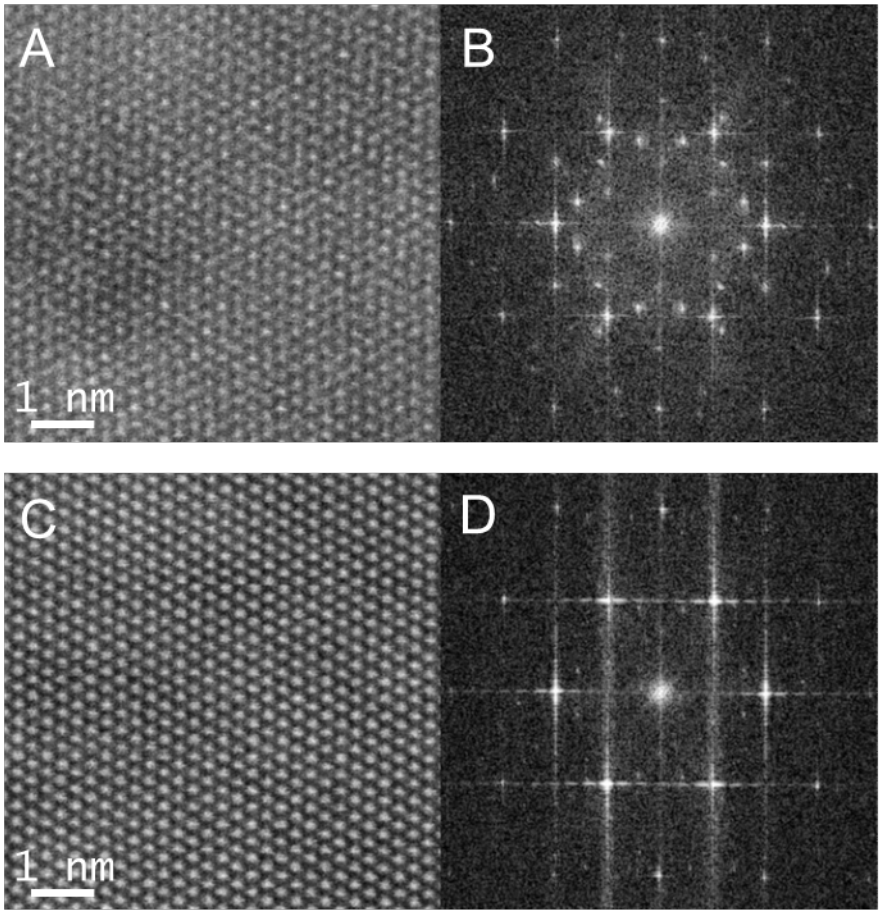

Supplementary Figure 13. HRSTEM through focus series showing encapsulating bismuthene structure. (**A**) HAADF-STEM through focus images of a bismuth crystallite along the [111] direction showing the bismuthene structure on the surface and (**B**) respective FFT. (**C**) Bi(111) structure at the interior of the crystallite and (**D**) FFT. Full dataset is shown in Supplementary Movie SM2.

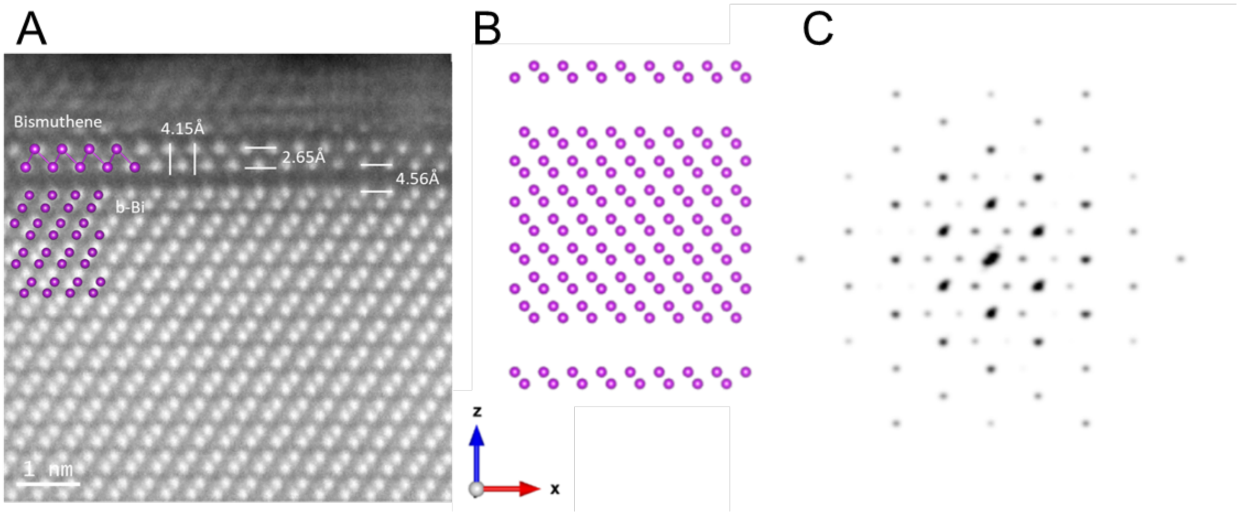

Supplementary Figure 14. Atomic resolved STEM and SAED simulation of lifted bismuthene patches. (**A**) ac-HRSTEM with separated individual monolayer bismuthene patch lifted from crystalline "bulk" resulting from a beam induced defunctionalization and subsequent structural relaxation. (**B**) Model for simulation of SAED pattern in (**C**).

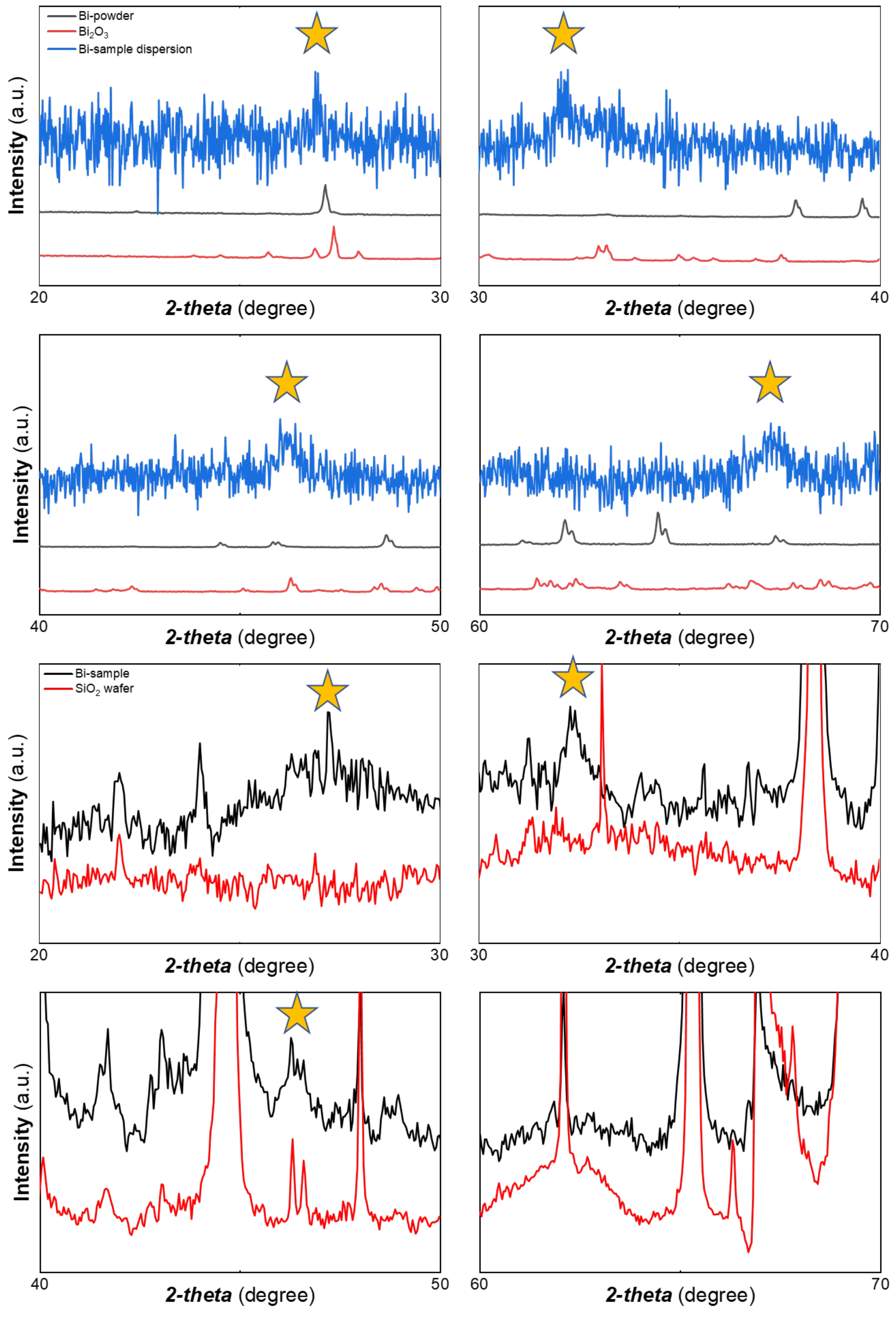

Supplementary Figure 15. Capillary XRD of the reaction mixture and comparison with Bi_2O_3 and Bi(0). The dispersed and surface XRD of the hybrid material, after the oxidation reaction of 4-bromo mandelic acid to 4-bromo benzoic acid, are mainly the same (compare blue lines above and black lines below), thus both measurements are comparable, which allows us to now compare with the surface XRD of bulk Bi_2O_3 and Bi(0). It can be seen that the XRD for the latter are completely different. The XRD have been split in portions of 2θ= 10º for a better visualization, since the XRD of the hybrid material in the dispersion is obviously very diluted.

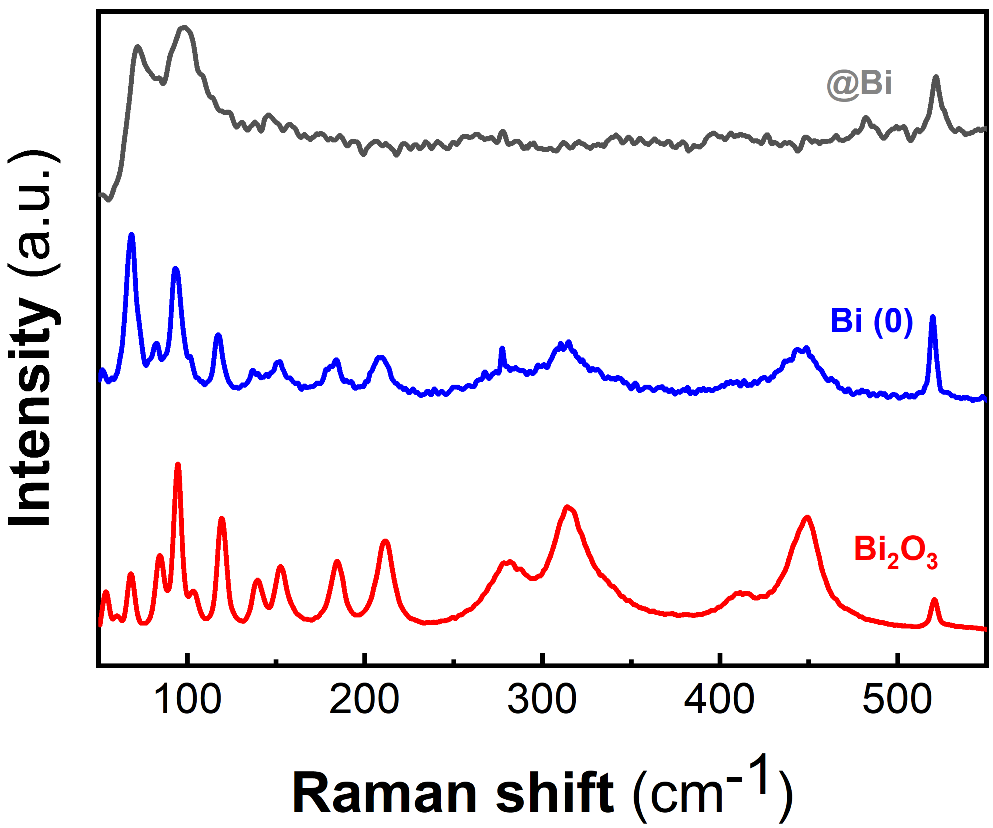

Supplementary Figure 16. Raman of the spent hybrid material, after reaction, and comparison with Bi_2O_3 and Bi(0). The Raman spectrum of the hybrid material, after the oxidation reaction of 4-bromo mandelic acid to 4-bromo benzoic acid, shows the main two peaks of the original material, and the spectra of Bi_2O_3 and Bi(0) are completely different.

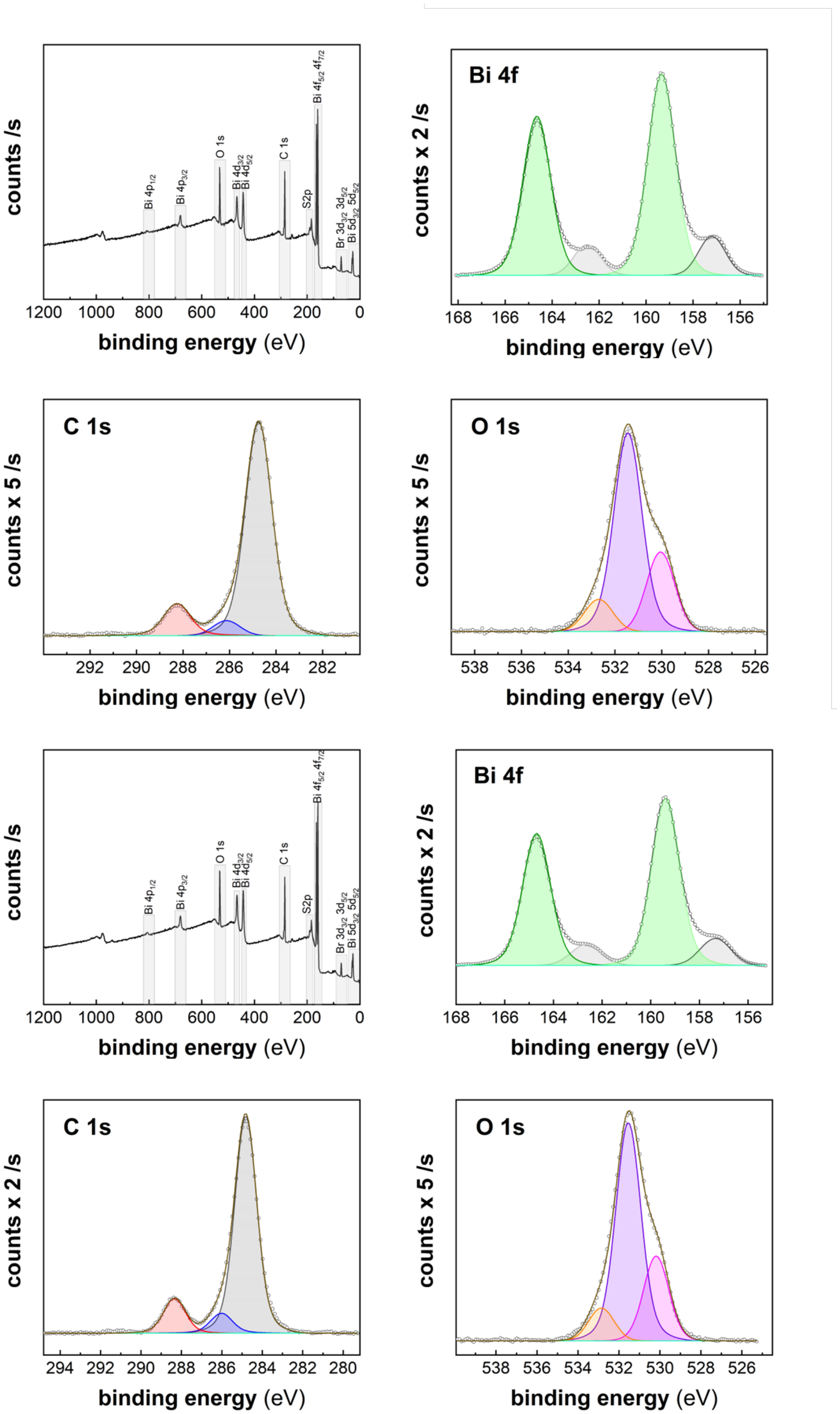

Supplementary Figure 17. XPS of the spent hybrid material. The XPS spectra of the hybrid material after the oxidation reaction of 4-bromo mandelic acid to 4-bromo benzoic acid, either at 200 (top) or 400 μm (bottom) spot sizes, shows the S 2p peaks together with the Bi 4f signals (see survey) and the complex deconvoluted signals for C 1s and O 1s, as in the original material.

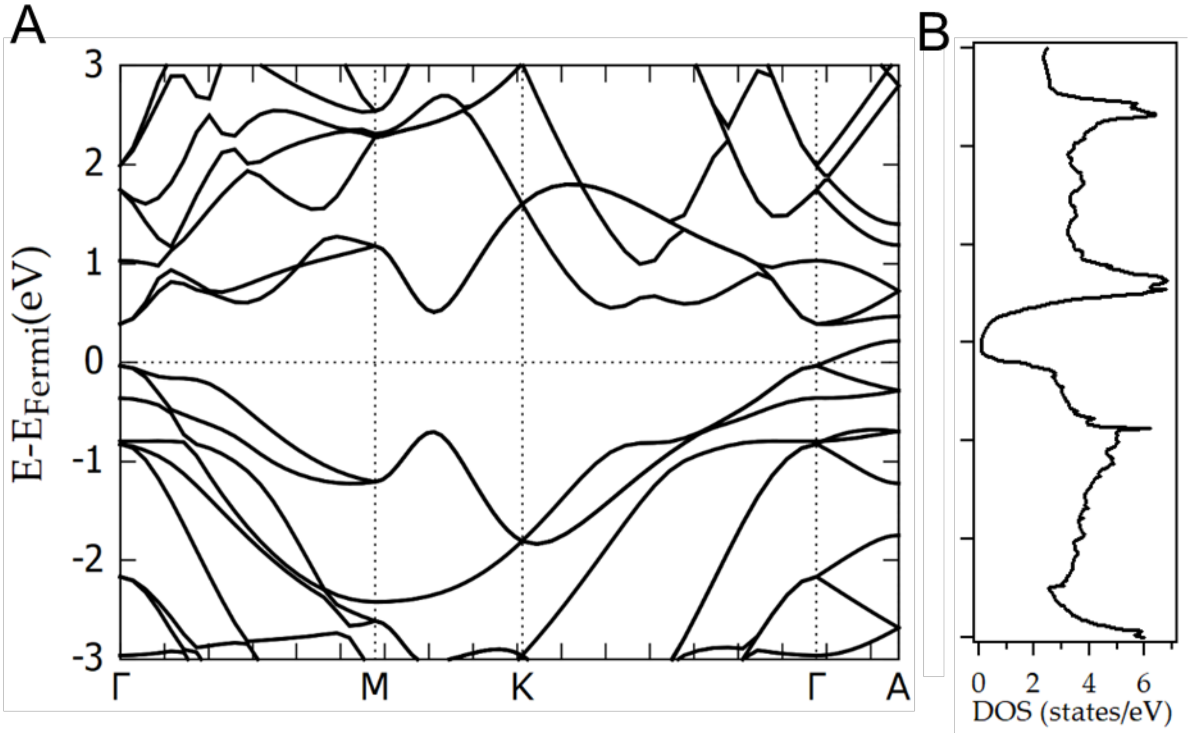

Supplementary Figure 18. Band structure of conventional β-bismuth. (**A**) Calculated band structure and (**B**) density of states of bulk bismuth structure.

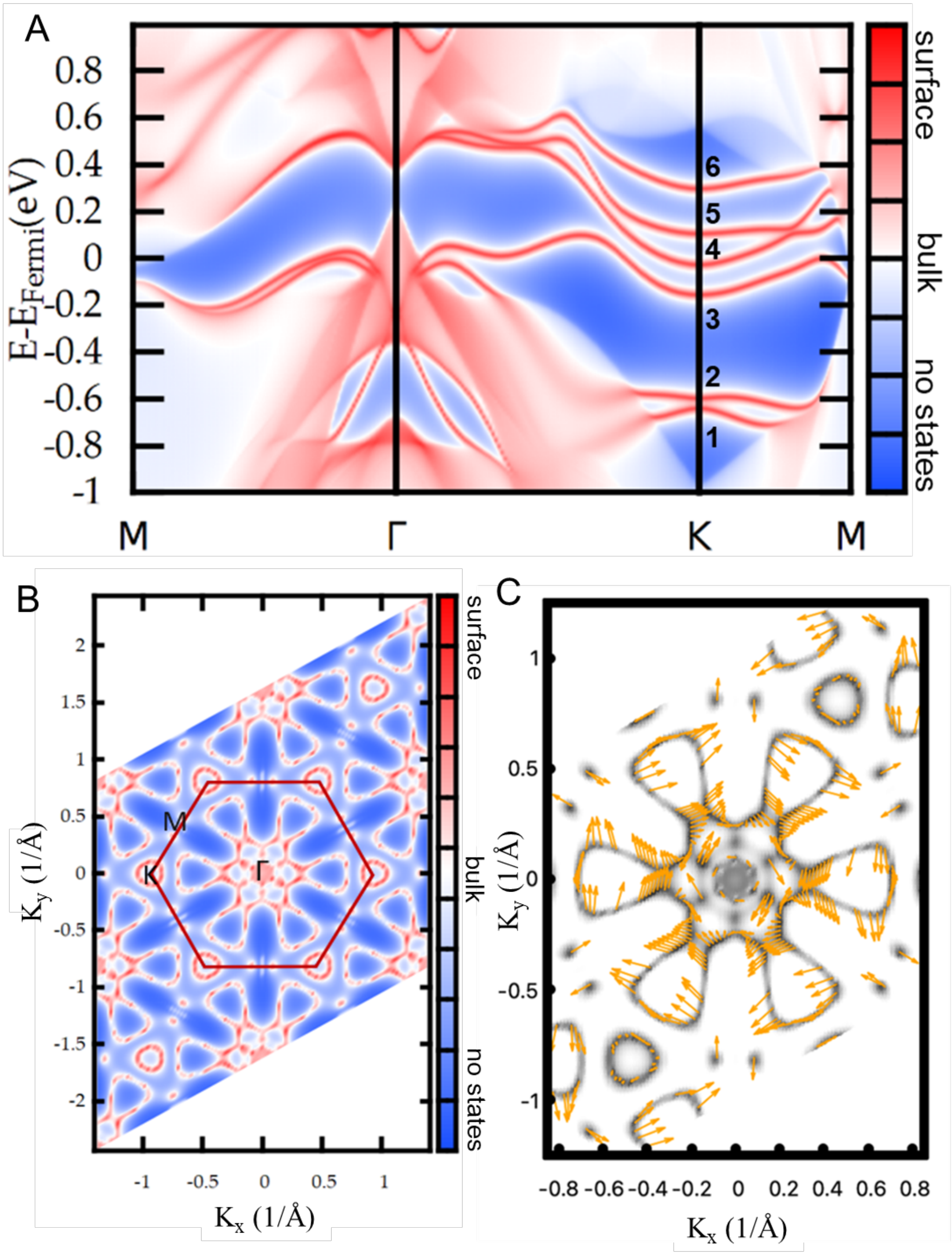

Supplementary Figure 19. Calculated surface states of half-layer terminated bismuth structure. (**A**) Surface spectrum, showing the distribution of states with six newly formed surface states around K. (**B**) Projected distribution of states in K-plane (Fermi surface). (**C**) Spin texture in K-plane

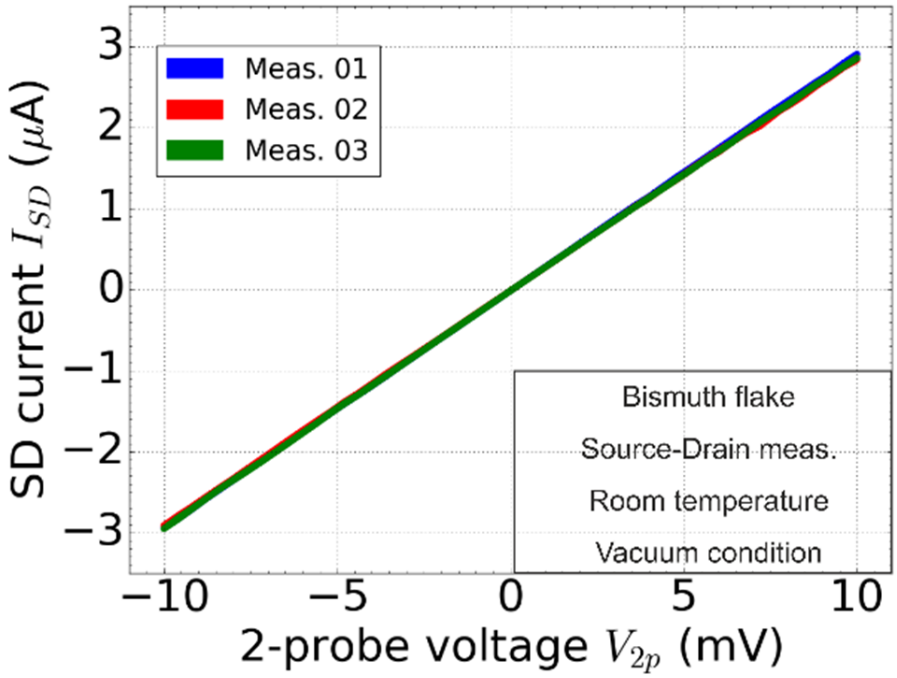

Supplementary Figure 20. 2 Probe measurement. Recorded I/V curves for 3 individual rt-transport measurements of the same device exhibiting ohmic behavior indicating a high reproducibility.

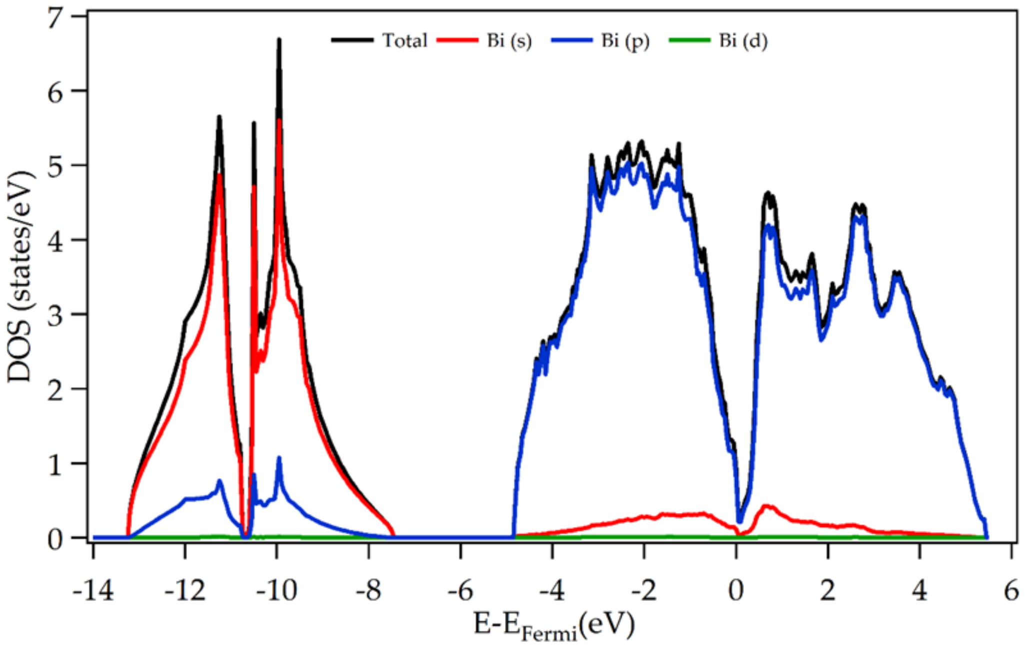

Supplementary Figure 21. Orbital resolved DOS for conventional β-bismuth. Total PDOS of Bi: *s*, *p* and *d* orbitals.

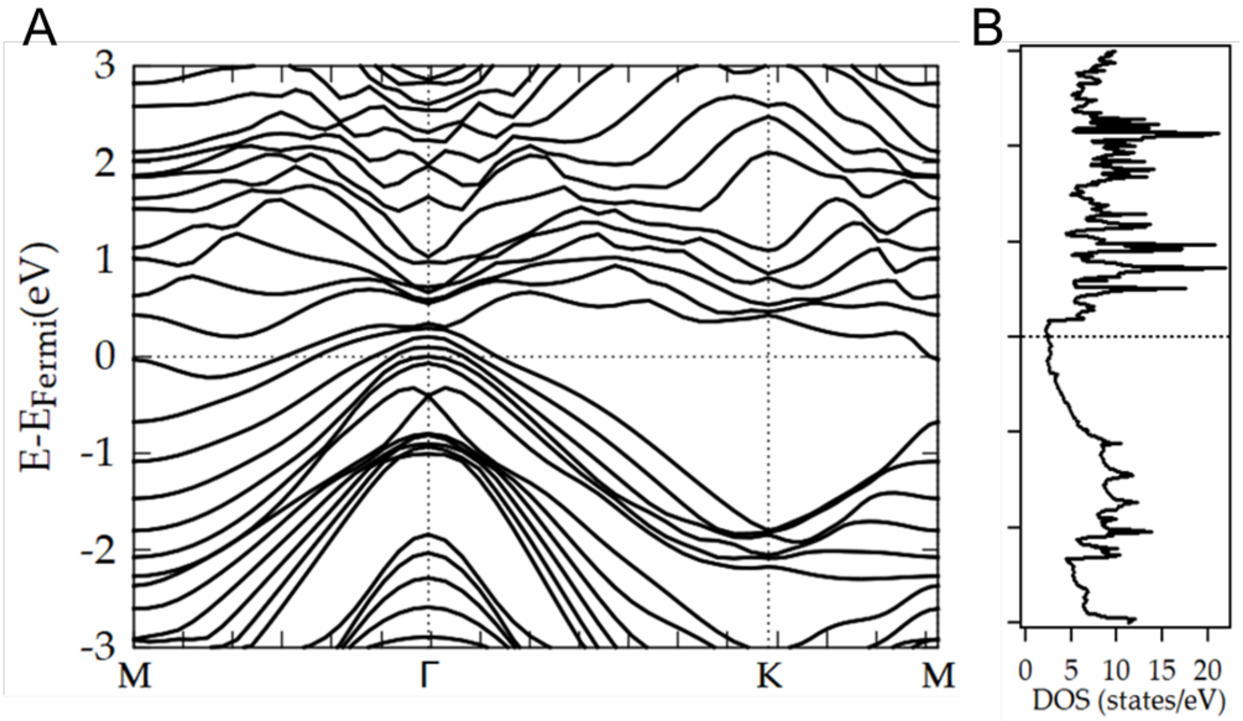

Supplementary Figure 22. **Band structure of six layer β-bismuth.** (**A**) Calculated band structure and (**B**) density of states of six-layer bismuth structure.

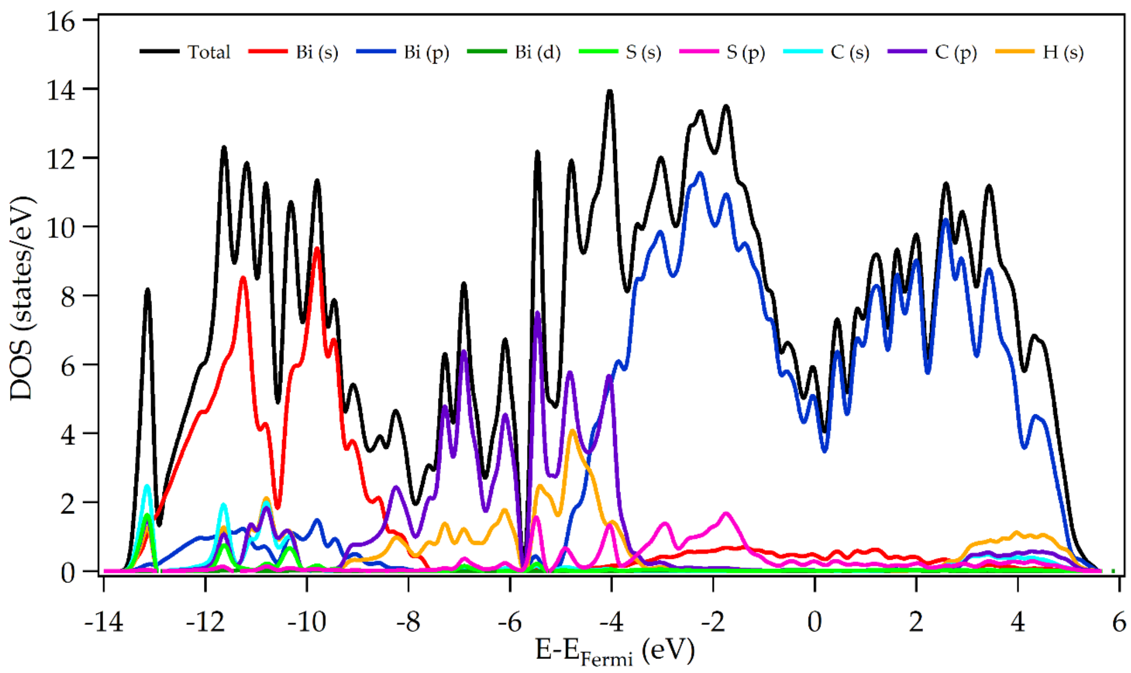

Supplementary Figure 23. **Orbital resolved DOS for bismuth hybrid material.** Orbital resolved density of states of hybrid structure.

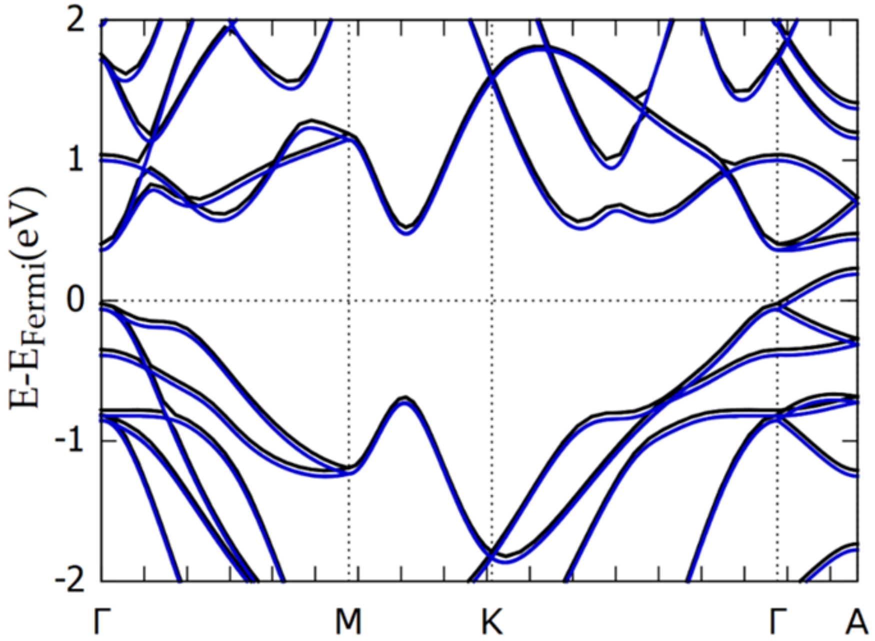

Supplementary Figure 24. Electronic band structure of bismuth hybrid. Calculated at PBE level (black) and MLWFs tight-binding model (blue).

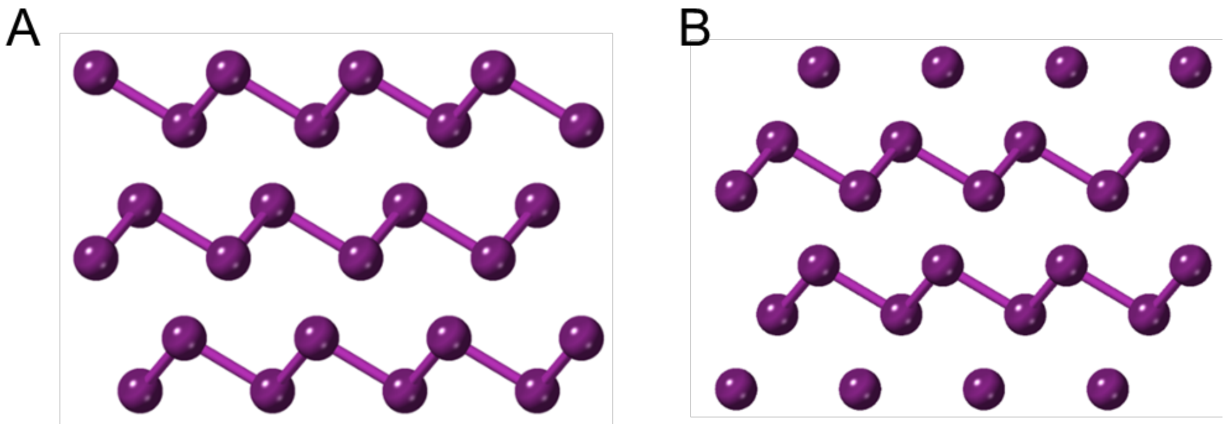

Supplementary Figure 25. Setup for structural models. (**A**) Conventional bismuth bulk structure, (**B**) half-layer terminated bismuth bulk structure.

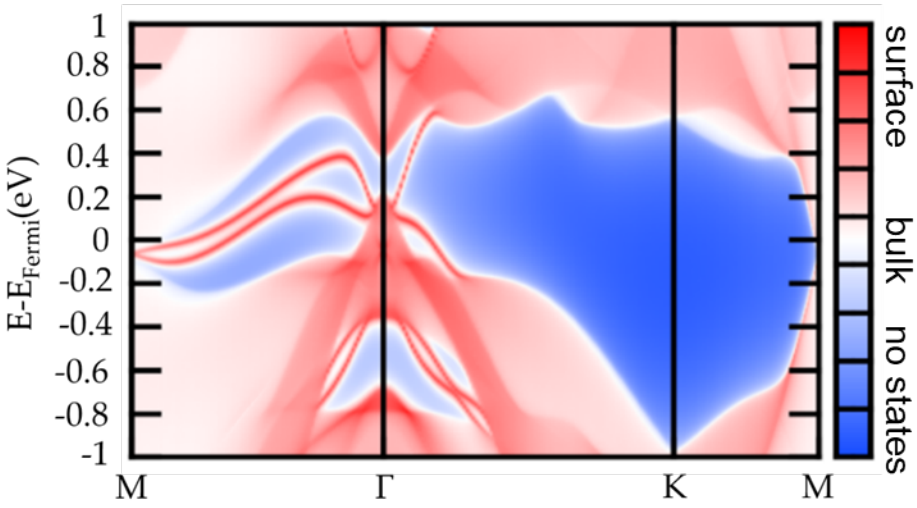

Supplementary Figure 26. Surface spectrum simulation of conventional β-bismuth. Contribution of different states to global band structure (res: surface states, white: bulk states, blue: no states) for 30 layer bismuth slab system.

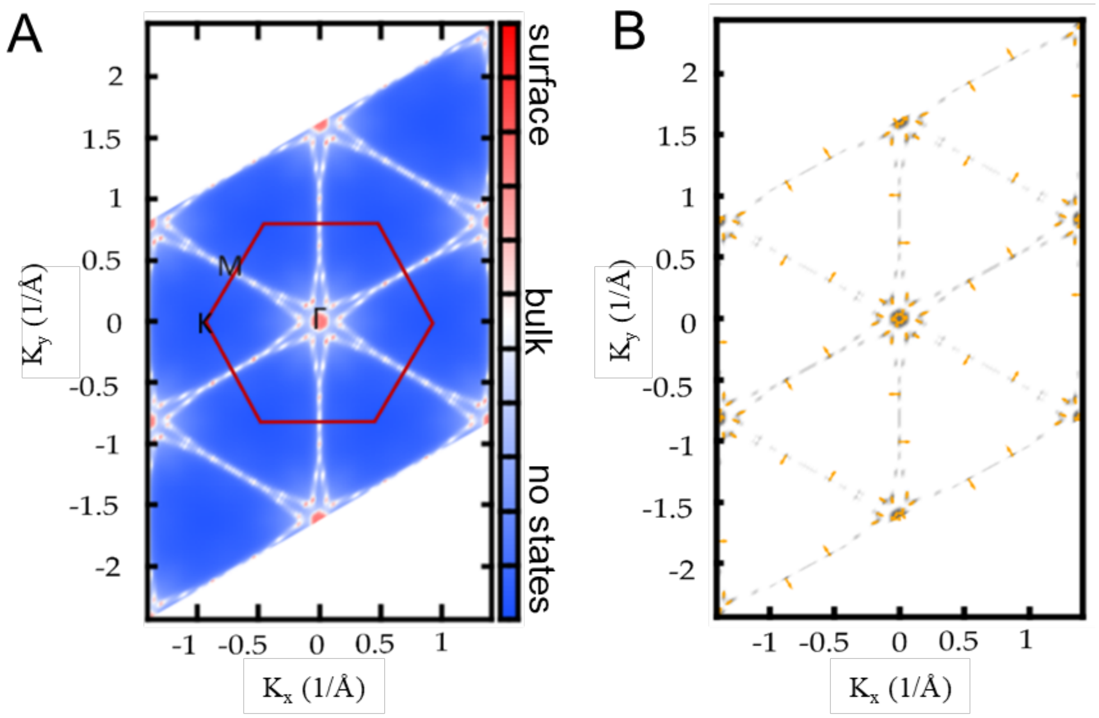

Supplementary Figure 27. Fermi surface of conventional β-bismuth. (**A**) Calculated Fermi surface with contributions from surface states (red) and bulk states (white). (**B**) Spin texture of a 30 layers bismuth slab system.

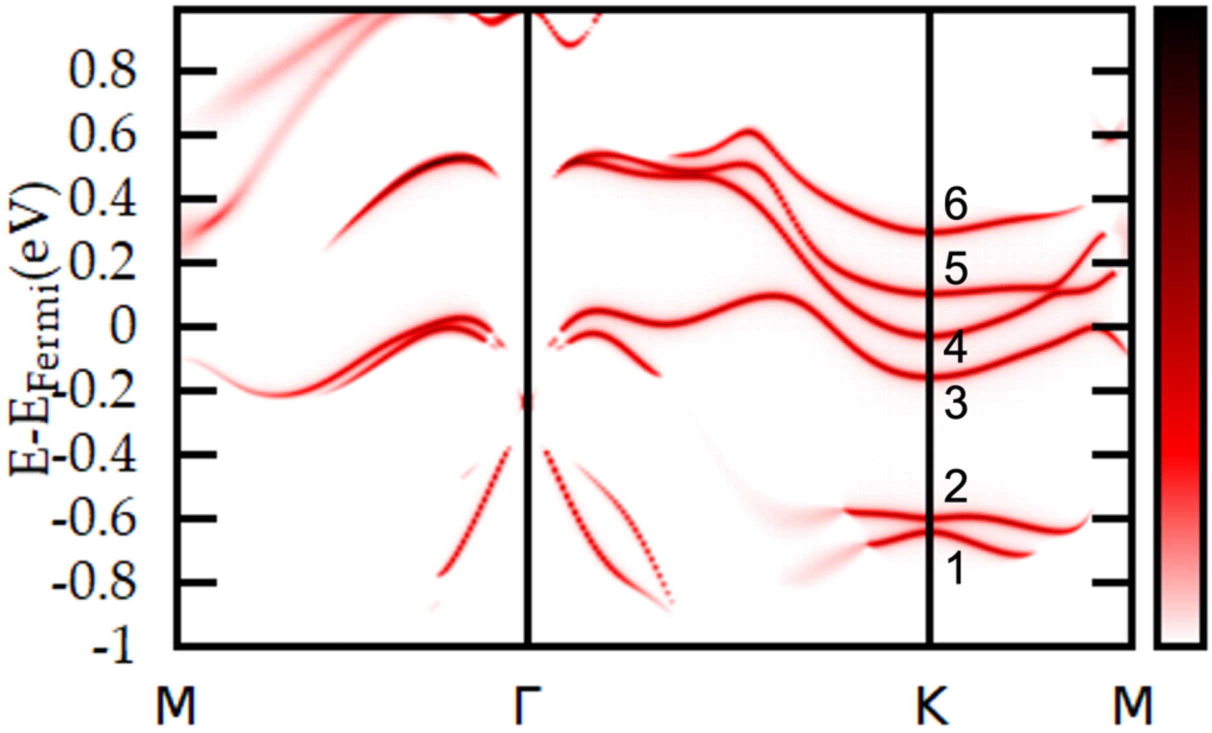

Supplementary Figure 28. Surface states of half-layer terminated bismuth structure. Only contributions to global band structure based on surface states shown in red. Numbering of newly established surface states at K as indicated.

Supplementary Table 3. Z_2 topological number and Z_2 invariants for six-time reversal invariant momentum planes in the half-terminated bismuth bulk structure model.

**Time reversal invariant momentum plane**	**Z_2 topological number**
$k_x = 0.0$	0
$k_x = 0.5$	0
$k_y = 0.0$	0
$k_y = 0.5$	0
$k_z = 0.0$	0
$k_z = 0.5$	0
**Z_2 invariants (ν_0; $\nu_1\nu_2\nu_3$)**	
(0; 000)	

Supplementary Movie SM1.

Video of redox reaction after addition of DDT. The color change from yellow to black can be followed. The reaction is terminated by quenching after the black suspension can be observed.

Supplementary Movie SM2.

Through focus series (ac-HAADF-STEM) of bismuth/ bismuthene hybrid obtained at 200 kV. Upon changing the defocus value different depths of the crystallite are sampled and allow for a discrimination between bulk and surfaces. Through focus series was acquired with a 30 mrad illumination semiangle and 1 nm defocus step.
